# Speaker-story mapping as a method to evaluate audiovisual scene analysis in a virtual classroom scenario

**DOI:** 10.3389/fpsyg.2025.1520630

**Published:** 2025-06-10

**Authors:** Stephan Fremerey, Carolin Breuer, Larissa Leist, Maria Klatte, Janina Fels, Alexander Raake

**Affiliations:** ^1^Audiovisual Technology Group, Technische Universität Ilmenau, Ilmenau, Germany; ^2^Institute for Hearing Technology and Acoustics, Rheinisch-Westfälische Technische Hochschule (RWTH) Aachen University, Aachen, Germany; ^3^Center for Cognitive Science, Rheinland-Pfälzische Technische Universität (RPTU) Kaiserslautern-Landau, Kaiserslautern, Germany

**Keywords:** immersive virtual environments, virtual reality, audiovisual scene analysis, audiovisual scene perception, dynamic binaural rendering, speaker-story mapping, classroom, task performance

## Abstract

This study explores how audiovisual immersive virtual environments (IVEs) can assess cognitive performance in classroom-like settings, addressing limitations in simpler acoustic and visual representations. This study examines the potential of a test paradigm using speaker-story mapping, called “audiovisual scene analysis (AV-SA),” originally developed for virtual reality (VR) hearing research, as a method to evaluate audiovisual scene analysis in a virtual classroom scenario. Factors affecting acoustic and visual scene representation were varied to investigate their impact on audiovisual scene analysis. Two acoustic representations were used: a simple “diotic” presentation where the same signal is presented to both ears, as well as a dynamically live-rendered binaural synthesis (“binaural”). Two visual representations were used: 360°/omnidirectional video with intrinsic lip-sync and computer-generated imagery (CGI) without lip-sync. Three subjective experiments were conducted with different combinations of the two acoustic and visual conditions: The first experiment, involving 36 participants, used 360° video with “binaural” audio. The second experiment, with 24 participants, combined 360° video with “diotic” audio. The third experiment, with 34 participants, used the CGI environment with “binaural” audio. Each environment presented 20 different speakers in a classroom-like circle of 20 chairs, with the number of simultaneously active speakers ranging from 2 to 10, while the remaining speakers kept silent and were always shown. During the experiments, the subjects' task was to correctly map the stories' topics to the corresponding speakers. The primary dependent variable was the number of correct assignments during a fixed period of 2 min, followed by two questionnaires on mental load after each trial. In addition, before and/or after the experiments, subjects needed to complete questionnaires about simulator sickness, noise sensitivity, and presence. Results indicate that the experimental condition significantly influenced task performance, mental load, and user behavior but did not affect perceived simulator sickness and presence. Performance decreased when comparing the 360° video and “binaural” audio experiment with either the experiment using “diotic” audio and 360°, or using “binaural” audio with CGI-based, showing the usefulness of the test method in investigating influences on cognitive audiovisual scene analysis performance.

## 1 Introduction

Classroom communication and comprehension are often challenging due to unfavorable listening conditions such as background noise and reverberation. Existing studies primarily focus on simple auditory tasks (see, e.g., Doyle, 1973; Rueda et al., 2004; Holmes et al., 2016; Röer et al., 2018), but actual classroom listening requires students to process complex, continuous speech in challenging listening environments. Understanding how varying acoustic and visual conditions affect comprehension in these settings is essential to support effective learning. Until now, existing paradigms have focused on relatively simple acoustic and visual representations (see, e.g., Spence and Driver, 1997; Koch et al., 2011; Lawo et al., 2014; Oberem et al., 2014, 2017, 2018; Barutchu and Spence, 2021; Fichna et al., 2021). In this study, the effectiveness of audiovisual virtual environments in assessing cognitive performance in classroom-like Immersive Virtual Environment (IVE) settings is investigated by creating complex visual and acoustic scenes in a controlled environment. To improve the validity of cognitive performance research in such environments, the realism of these paradigms needs to be progressively increased, especially in terms of the visual scene complexity. It is hypothesized that a more sophisticated audiovisual representation, and hence a more medially rich representation of the virtual environment, has a positive impact on task performance.

To do so, in this article the “audiovisual scene analysis test paradigm” originally developed by Ahrens et al. (2019) and Ahrens and Lund (2022) is adapted and expanded to assess its suitability for analyzing audiovisual scene analysis in a virtual classroom scenario. To maintain consistency with existing research, it was intentionally decided to keep the original naming convention of the paradigm. In the paradigm originally developed by Ahrens et al. (2019), multi-speaker scene perception has been investigated regarding auditory and visual information. Twenty-one speakers were depicted as schematic avatar silhouettes positioned semi-circularly around the listener, ranging from –90° to 90° in 30° steps. In each trial, 2–10 speakers simultaneously read stories from different locations, without visual feedback of which of the speakers were active, while visually all 20 possible speakers were always shown. During the actual perception test, six normal-hearing subjects, wearing an HTC Vive Pro HMD, were asked to match the stories to the corresponding visual locations of the speakers, with the speakers and their positions being randomly assigned in each trial. Authors concluded that participants were able to accurately analyze scenes with up to six speakers. However, when more speakers were added to the scene, azimuth localization accuracy declined, while distance perception remained consistent regardless of the number of speakers.

While classical frontal-teaching classroom settings typically involve only a few simultaneously active speakers, interactive classroom settings and realistic classroom activities, such as resulting group work, discussions, collaborative exercises, and other interactive learning formats, often create situations where several conversations happen simultaneously, resulting in multiple simultaneous speakers in one room. In these situations, it is essential to focus on the content spoken by a single speaker, known in literature as the cocktail party effect (Cherry, 1953; Bronkhorst, 2000; Shinn-Cunningham, 2008; Bronkhorst, 2015). Such scenarios may occur in real classroom environments, for example, when multiple smaller groups are talking in close proximity. Understanding cognitive performance in these contexts, even in extreme conditions such as having 10 simultaneously active speakers, is crucial, even though such scenarios might not occur every day in real classrooms. Hence, this study proposes a psychometric method to investigate audiovisual interaction effects by measuring cognitive performance in scenarios with good speech intelligibility, aiming to systematically evaluate cocktail party settings. To achieve this, the number of simultaneous speakers was intentionally and systematically increased, an approach previously demonstrated to be an appropriate independent variable by Ahrens et al. (2019) and Ahrens and Lund (2022). Further, the paradigm proposed in this study systematically extends the visual aspects of the paradigm initially suggested by Ahrens et al. (2019), which were previously underrepresented due to the use of a rather simple visual scene featuring semi-transparent avatar silhouettes. The proposed paradigm enables a deeper understanding of psychometric aspects of audiovisual paradigms, more specifically the audiovisual interplay through a realistic and controlled IVE setting, which is crucial for moving toward an even more realistic and child-appropriate scenario in the future, such as collaborative group work and discussion settings.

In Fremerey et al. (2024), a modified version and implementation of the original paradigm by Ahrens et al. (2019) was published, referred to as AVT-ECoClass-VR.^1^ It features two different visual instances for different audiovisual scenarios, namely 360° video and computer-generated imagery (CGI), both including 20 different speakers and two implementations for dataset playback. The 360° video scenario consists of individually recorded 360° videos of speakers, embedded together within a 360° image scene. The CGI scenario represents a computer-generated classroom environment created using SketchUp Make 2017, including 3D-scanned avatars of the same individuals recorded in the 360° video scenario. Thus, each speaker is represented both as a 360° video recording and as a 3D-scanned avatar. Hence, for auditory cognition research, the visual representations of persons in both virtual classroom settings can be combined with different acoustic presentations of the speech signals initially recorded together with the 360° video representations. In the research presented in this study, to investigate which factors are having an influence on dependent variables like the perceived task performance, a simplified but easily accessible diotic presentation via headphones is compared to a dynamic binaural rendering.

In this way, the goal of the study is to develop a psychometric method to investigate audiovisual interaction effects by varying the complexity of the audiovisual scene, with an initial focus on measuring cognitive performance by adjusting the number of simultaneously active speakers, ranging from 2 to 10, in an IVE setting with good speech intelligibility. The audiovisual scene dataset and tools made available by the authors in Fremerey et al. (2024), building on Ahrens et al. (2019), increase the number of available speakers and add both a 360° and a CGI visual representation.

This study systematically addresses the question of the extent to which manipulating the auditory and visual presentation of an IVE influences factors such as task performance, mental load, and user behavior in the context of the speaker identification task. More specifically, in this article, differences between the two classroom scenario-like IVEs, 360° video and CGI, in conjunction with the two audio settings “diotic” and “binaural,” are investigated regarding cognitive performance, user behavior (cf. Fremerey et al., 2018), task load (cf. Georgsson, 2019), presence (cf. Schubert et al., 2001), and simulator sickness (cf. Kennedy et al., 1993). To this end, a series of three subjective tests is conducted, where subjects are instructed to map simultaneously told stories to the respective visual representations. Hence, this study contributes to the broader research topic on multisensory perception by examining how audiovisual coherence and the characteristics of different experimental conditions influence cognitive performance in IVE settings.

Three subjective experiments were conducted with different combinations of the two acoustic and visual conditions: The first experiment used 360° video but with binaural audio. The second experiment combined 360° video with diotic audio. The third experiment used the CGI environment with binaural audio. During the experiments, it became clear that the difficulty level of the 360° diotic experimental condition was already high enough, despite good lip-sync quality. Therefore, we decided not to explicitly include an additional CGI diotic experimental condition, especially since technical limitations of the IVE would not have allowed lip-sync of comparable visual quality to the 360° video. A CGI diotic experimental condition without lip-sync would have theoretically also been possible but was not included, as it would have provided no localization information through either the auditory or visual channel. In such a scenario, participants would have relied more on guessing than on actual knowledge for speaker-story assignments. In summary, this led us to decide against integrating any CGI diotic experimental condition into our experiments.

The goal is to answer the following research questions, while in brackets the respective dependent variables used to investigate the specific research question are mentioned:

- RQ1: How does the task performance correlate with the results of Ahrens et al. (2019) for different levels of the total number of stories presented simultaneously, across the two visual IVE representations (360° video vs. CGI) and audio conditions (binaural vs. diotic)? (Dependent variables: correctly assigned stories, total time needed)
- RQ2: How do the total number of stories presented simultaneously, the audio condition (binaural vs. diotic), and the visual IVE representation (360° video vs. CGI) impact task performance and mental load? (Dependent variables: correctly assigned stories, total time needed, NASA RTLX results, listening effort)
- RQ3: Is the user behavior, in terms of head movement, different across audio conditions (binaural vs. diotic) and visual IVE representations (360° video vs. CGI)? (dependent variables: proportion of time spent watching active speakers, total yaw degrees explored, total number of yaw direction changes)
- RQ4: What differences in constructs related to Quality of Experience (QoE) for IVEs, such as simulator sickness and presence, exist across different audio conditions (binaural vs. diotic) and visual IVE representations (360° video vs. CGI)? (dependent variables: simulator sickness, presence questionnaire results)

Based on the research questions, the hypotheses of this study are presented as follows:

- H1: It is hypothesized that task performance in both visual IVE representations (360° video and CGI) shows a strong correspondence with the performance observed in the study by Ahrens et al. (2019), regardless of whether the audio condition is binaural or diotic.
- H2: While a general decline in task performance and an increase in mental load with a higher number of simultaneously presented stories is expected, it is hypothesized that these effects will be more visible in the diotic audio condition compared to the binaural condition and in the CGI representation compared to the 360° video.
- H3: It is hypothesized that head movement patterns (e.g., proportion of time spent watching active speakers, total yaw degrees explored, total number of yaw direction changes) will differ between binaural and diotic audio conditions, as well as between 360° video and CGI representations.
- H4: It is hypothesized that simulator sickness levels (before/after the experiment) will not differ between audio conditions (binaural vs. diotic) or visual IVE representations (360° video vs. CGI). However, differences are expected in the sense of presence, with higher levels of presence reported for binaural audio conditions and 360° video representations.

## 2 Background

The following provides an overview of the existing state-of-the-art research on the design of audiovisual virtual environments to investigate cross-modal perception and task performance in established cognitive tasks.

Stecker (2019) emphasize the potential benefits of Immersive Virtual Environments (IVEs) for assessing auditory performance, as they improve multisensory consistency, can bring the real world into the lab, enable natural multidimensional tasks, and enhance the engagement of subjects. Furthermore, as stated in Owens and Efros (2018), the visual and auditory components of a video should be jointly modeled through a combined multisensory representation. Based on these requirements to improve the validity of state-of-the-art research, the present study aimed to increase the complexity of virtual audiovisual scenes and to investigate their impact on perceived mental load as well as presence.

Virtual reality (VR) has gained significant popularity in cognitive psychology research over the past two decades (see Foreman, 2010; Schnall et al., 2012), providing innovative methods to examine how the features of these virtual audiovisual environments influence auditory perception. Other studies that specifically evaluated speaker identification as a task are, for example, the study by Stecker et al. (2018). In this study, an experiment with six participants was conducted to measure co-immersion in virtual auditory scenes using a spatial localization and speaker identification task. The authors conclude that listeners can distinguish the reverberant characteristics of multiple simultaneous speakers in a complex auditory scene when visual, auditory, and dynamic information about the talkers' locations is provided. Another study is the one by Josupeit and Hohmann (2017), where speaker identification, speech localization, and word recognition were modeled for a multi-speaker setting. The model is able to extract salient audio features from a multi-speaker audio signal and use a classification method that matches these features with templates from clean target signals to determine the best target. In the study by Rungta et al. (2018), the influence of reverberation and spatialization on the cocktail-party effect was evaluated for a multi-speaker IVE. The cocktail party effect was introduced by Cherry (1953) and further investigated in, e.g., Bronkhorst (2000), Bronkhorst (2015), and Shinn-Cunningham (2008). It describes the need for spatial attention to distinguish relevant acoustic information from multiple competing acoustic sources, such as several simultaneous conversations at a party. In adverse cases, classroom communication and comprehension could be comparable to the cocktail party effect.

With the aim to investigate differences between binaural and diotic listening, Rungta et al. (2018) used diotic audio and two different methods of spatialization: stereo using vector-based amplitude panning and binaural convolving a generic KEMAR head-related impulse response (HRIR) (cf. Gardner and Martin, 1995) with the room impulse response giving the binaural room impulse response (BRIR) for the listener. In all cases, the audio was played back through the integrated headphones of the Oculus Rift CV1 HMD. The authors found that spatialization had the highest impact on target-word identification performance, as binaural listening outperforms diotic listening and has been shown to be robust with respect to the arrangement of distractor stimuli. Increased reverberation negatively affects speech intelligibility by decreasing the robustness of diotic and binaural cues, with the effect becoming more visible when reverberation is higher.

Another study by Gonzalez-Franco et al. (2017) aimed to investigate how audiovisual cues affect selective listening using virtual reality and spatial audio. To do so, participants were exposed to an information masking task with concurrent speakers. To this aim, wide-field-of-view stereoscopic video and audio recordings were made. The resulting recordings were rendered in VR as 185° stereoscopic videos. The results show that asynchronous visual and auditory speech cues, particularly mismatched lips and audio, significantly impact comprehension and auditory selective attention, emphasizing the importance of visual cues in multisensory integration. In relation to this, Vollmer et al. (2023) conducted an audiovisual serial recall experiment with 13 participants. Authors found that for audiovisual speech recordings, the recall performance is significantly better for presentations with auditory stimuli than for visual-only presentations. However, the audiovisual representation did not significantly improve the recall performance.

A complementary dataset with 360° video is described in Kishline et al. (2020). It presents a database from five speakers recorded as anechoic audio speech samples with stereoscopic and omnidirectional video. This study provides tools and resources that shall enable the simulation of scenarios with up to three speakers positioned at various azimuthal and depth locations in an IVE. It is noted that the initially publicly available dataset can no longer be found under the link given in Kishline et al. (2020).

In relation to the previously described study, Fichna et al. (2021) aimed to investigate the effects of acoustic scene complexity and visual scene representation on auditory perception in IVEs. To do so, authors used a combination of an HMD and a 3-dimensional 86-channel loudspeaker array, assessing five psychoacoustic measures: speech intelligibility, sound source location, distance perception, loudness, and listening effort. The subjective test involved twelve listeners, who performed a speech perception and assessment task in both echoic and anechoic virtual environments. During the task, the same fixed order was followed: after presenting the target and maskers, participants repeated the sentence of the target speaker, named the position of the target, rated the loudness, and rated the listening effort. Target distances, number of maskers, and reverberation conditions were varied. Results showed that reverberation and the number of interfering sound sources significantly affected the five psychoacoustic measures, contributing to scene complexity. Wearing an HMD did not substantially alter performance, indicating that the setup allows for realistic testing of auditory perception. The study emphasizes the ecological validity of IVEs and their potential for comparing virtual and real-world acoustic environments.

Another study by Slomianka et al. (2024) investigated how eye and head movements are influenced by the complexity of audiovisual scenes, by factors such as reverberation and the number of concurrent speakers. To analyze these, the authors conducted a test with thirteen normal-hearing participants engaged in a speech comprehension and localization task, using the original version of the paradigm by Ahrens et al. (2019). The results show that increased scene complexity delays initial head movements, extends the search period, and leads to more gaze shifts and less accurate final head positioning when identifying the target speaker.

## 3 Materials and methods

The following section describes the test setup for the three tests, including test stimuli and methods, details about the participants and pre-screening, instructions, tasks, independent and dependent variables, and information about the dataset and technical setup.

### 3.1 Test stimuli and test method

Within this research, three different perceptual tests have been carried out, using the two different Unity IVEs 360° videos and CGI published in Fremerey et al. (2024). Two different audio settings and two visual representations for classroom-like IVE settings have been tested: 360° video and CGI, where the 360° reference visual condition with intrinsic lip-sync of the comprised audio recordings was conducted once with binaural and once with diotic audio. As detailed in Fremerey et al. (2024), the IVEs were developed to enhance the complexity of the audiovisual scenes to more accurately simulate a typical classroom environment. The virtual classroom was equipped with 20 chairs and speakers arranged in a circle with a diameter of 2.6 m. The 360° video and CGI visuals in the AVT-ECoClass-VR dataset are accompanied by 10 – reflecting the number of different stories – single-channel speech recordings per speaker, which can be positioned at various spatial locations within the virtual scene, here located on the speakers. This is done using VAUnity,^2^ a Unity package of the Virtual Acoustics v2022a auralisation software by IHTA, RWTH Aachen University (2024).

The first two experiments, “360° binaural” and “360° diotic,” have been carried out using the 360° video version, while the third experiment, “CGI binaural,” used the CGI version of the AVT-ECoClass-VR IVE. The first and third experiments used binaural audio, and the second experiment used diotic audio. To ensure consistency with the other two tests, the diotic audio setup for the second experiment was also performed using the VAUnity package, with the *MyAmbientMixer* renderer implemented to make the sound audible without spatialization. The live-tracked dynamic binaural audio setup for the first and third experiments was performed using VAUnity, using the *MyBinauralFreeField* renderer with a 1*x*1° HRTF of the ITA artificial head.

### 3.2 Participants and pre-screening

The subjective experiments have been approved by the ethics committee of the Technische Universität Ilmenau, cf. the positive ethics vote from 14 March 2023. In the “360° diotic” test, 25 subjects participated (nine female, 16 male, mean age 29.5); in the “360° binaural” test, 36 subjects (10 female, 26 male, mean age 29.8); and in the “CGI binaural” test, 34 subjects (18 female, 16 male, mean age 33.6). To avoid potential learning effects, each participant was limited to participating in only one of the three experiments. Subjects were recruited from the university student and staff body via email lists. Due to the fact that the stories were only available in German, an inclusion criterion for all experiments was that the participants had to have German language proficiency at the native speaker level. A further criterion was normal hearing ability within 20 dB. This was tested before each experiment using an Oscilla Audiometer USB100 with frequencies between 125 and 8,000 Hz, using the “automatic 20 dB test” functionality of the software AudioConsole v2.4.8 connected to the audiometer. In addition, subjects of all experiment groups were tested for visual acuity and color vision using Snellen (20/25) (cf. Pro Visu Foundation, 2023) and Ishihara (cf. Ishihara, 2009) charts.

### 3.3 Instructions

After the subjects gave their informed consent, they were introduced to the test procedure using an adapted version of the publicly available AVrateVoyager tool published by Göring et al. (2021), which was used during all experiments to answer the questionnaires later explained in Section 3.4.3. Afterwards, the operation of the IVE with the HTC Vive controller was explained. Then, the interpupillary distance of the HMD was adjusted to enable comfortable viewing of the IVEs. Afterwards, both the correct fit of the headphones and the HTC Vive Pro 2 HMD were checked. During the experiments, subjects had the task of correctly assigning the stories to the corresponding active speakers. They were allowed to revise and reassign their choices as needed. During the speaker identification task, participants were instructed to remain seated but were free to rotate their head and controller in any direction. Once participants completed the task or 2 min had passed, they were asked to remove the HMD and headphones and complete the questionnaires.

### 3.4 Task

In a subsequent training session with four trials, subjects were familiarized with the test procedure, the questionnaires, as explained in Section 3.4.3, and the controller interaction. The task of the subjects in all three experiments was to correctly map the stories to their corresponding speakers as fast as possible, using an HTC Vive 2.0 controller. To do that, subjects had 2 min of time. They were instructed to press the controller's menu button when they completed the speaker-story assignments, resulting in the termination of the IVE. The IVE session ended either when 2 min expired or when participants pressed the controller's menu button. In both cases, after subjects exited the IVE, the speaker-story mappings were saved, the IVE was terminated, and the subjects were asked to remove headphones, controller, and HMD.

#### 3.4.1 General aspects

**Figure 1:**
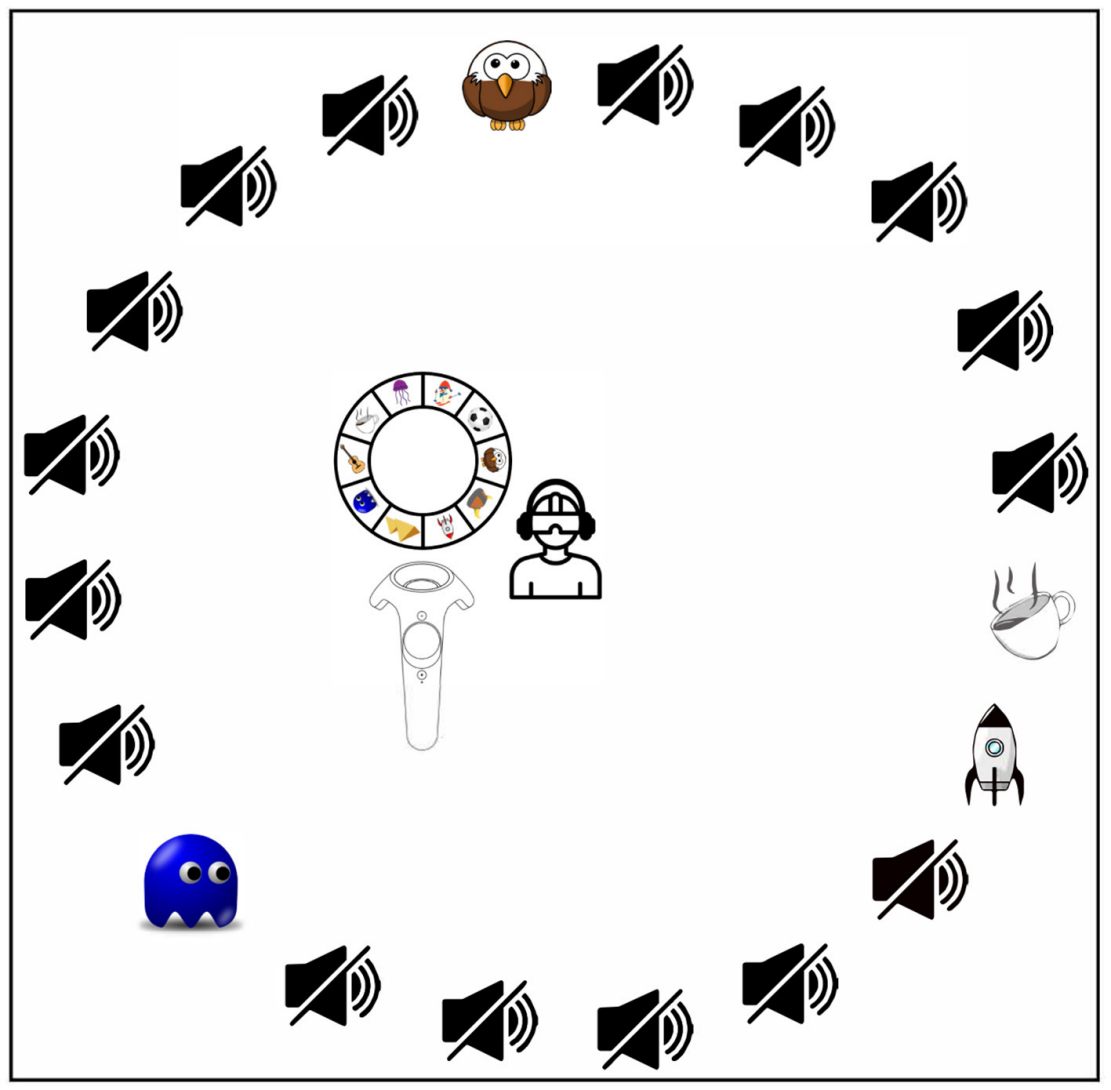
Visualization of the task used in all three experiments. The listener is located in the middle of a circle of 20 chairs with 20 speakers, of which 10 of them can potentially simultaneously each read out one story out of 10 possible stories. On the left of the listener, the controller with the interaction wheel is shown; for more details, see the paper text and Figure 2. The silent loudspeaker symbol represents a silent speaker, while the symbol refers to the story narrated by one of the 20 possible speakers. Here, an exemplary trial with four simultaneously active speakers, each narrating different stories, and 16 silent speakers is presented.

**Figure 2:**
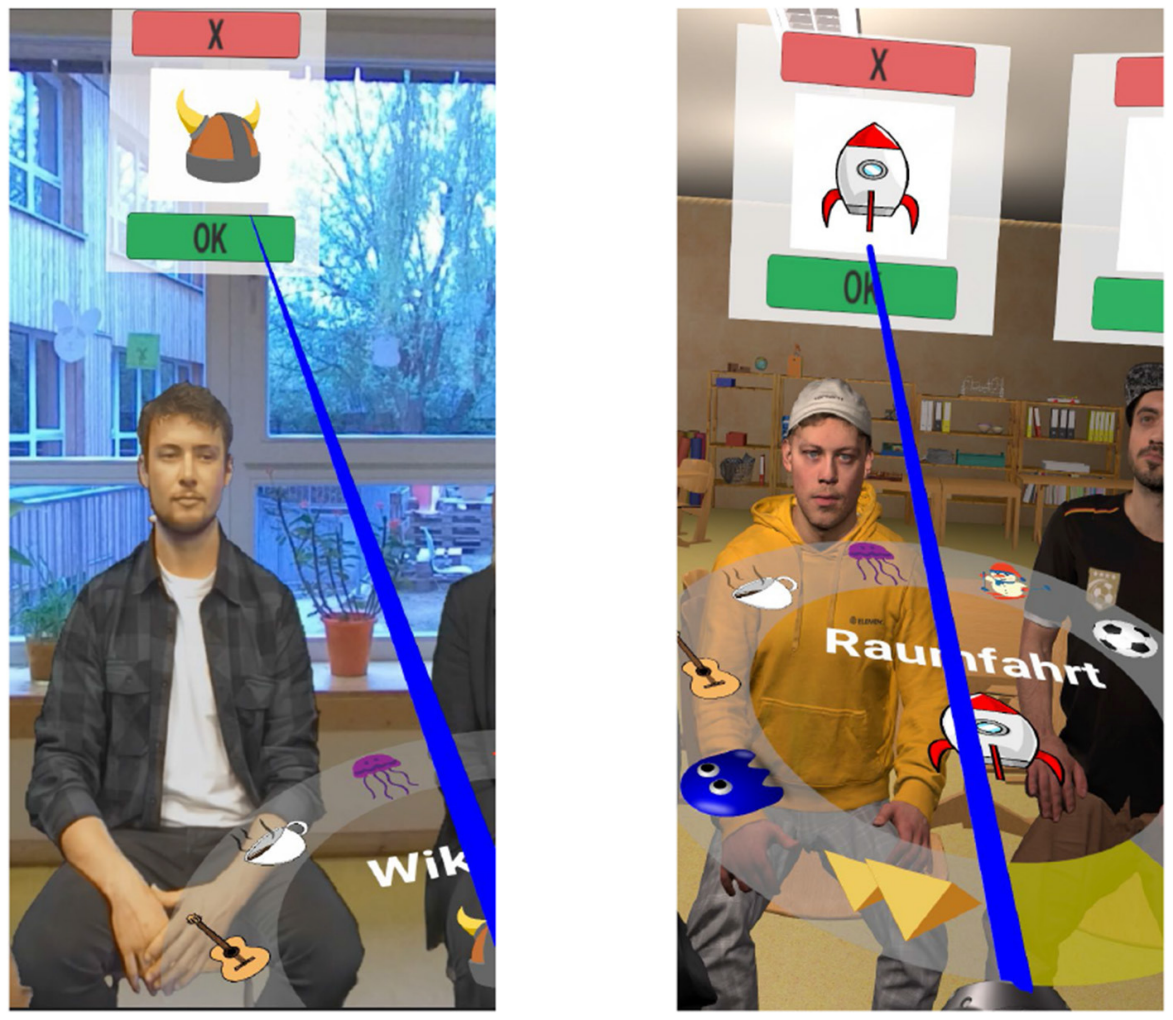
Speaker-story mapping system in the 360° IVE **(left)** and the CGI IVE **(right)**, using an HTC Vive 2.0 controller and the interaction wheel.

A general visualization of the task in all three experiments is shown in Figure 1. During the experiments, participants were placed on a rotating chair in the middle of the circle of 20 chairs, with the headset and headphone cables hanging from a hook above, as previously done by Agtzidis et al. (2019). All 20 possible speakers, 10 female and 10 male speakers recruited from the university student body, were always shown. This setup enabled them to rotate the chair freely without being hindered by cables, which might otherwise have caused them to avoid head and chair rotations and thus to orientate toward rearward speakers. In one hand, they hold the controller with the interaction wheel sticking above it. Subjects were asked to correctly assign the stories to the corresponding speakers by first selecting one of a total of 10 unique symbols representing the topics of the 10 possible stories, using the interaction wheel shown in Figure 1. The assignment of a story to a speaker was done using a blue ray appearing at the end of the controller and pointing to the green “OK” button while simultaneously clicking the controller's trigger button. The latter is more specifically shown in Figure 2, on the left for the 360° video IVE and on the right for the CGI IVE. Subjects were also allowed to remove a given assignment using the same principle explained above, now with the red “X” button, with the removed story appearing again on the interaction wheel.

The 10 possible stories were taken from Ahrens et al. (2019) and modified (cf. Fremerey et al., 2024) to extend them to 120 s, which was the maximum allowed time to complete the task. The story topics included jellyfish, skiing, soccer, birds, Vikings, outer space, pyramids, Pac-Man, guitar, and coffee, each providing general facts about its subject. The complete German versions of the stories were previously published in Fremerey et al. (2024).

#### 3.4.2 Training

In the training session, subjects experienced the respective IVE with one, two, five and 10 stories presented simultaneously, while the remaining speakers kept silent. The order of the number of simultaneously active speakers has been kept constant across all training sessions for all experiments to not directly overwhelm subjects with, e.g., 10 active speakers in the first training trial. To familiarize subjects with not only the handling of the IVE, subjects also needed to fill in the questionnaires after each trial. This has been done to provide participants with an impression of how the subjective test procedure would look and also to indicate the kind of difficulty they can expect in terms of speaker-story mapping.

#### 3.4.3 Questionnaires

In all three subjective tests, once before the training session, subjects were asked to answer the Simulator Sickness Questionnaire (SSQ) (Kennedy et al., 1993), consisting of 16 questions regarding different symptoms of simulator sickness on a discrete scale from 0 to 3. The SSQ is widely used in literature for assessing simulator sickness in IVE settings; see, e.g., Lin et al. (2002), Singla et al. (2017), and Sevinc and Berkman (2020). The SSQ was administered to obtain a baseline from the subjects regarding their general feelings before the experiment. Moreover, the three different IVE instances can only be utilized to evaluate audiovisual scene analysis in a virtual classroom scenario if they do not induce a significant level of simulator sickness. Furthermore, at the beginning of the test session, subjects were asked to fill in the German version of Weinstein's noise sensitivity scale (Zimmer and Ellermeier, 1997) once per test, consisting of 21 questions on a discrete scale from 0 to 4.

In all three experiments, after each of the nine trials, subjects left the IVE and were asked to provide ratings on five scales regarding effort, frustration, mental and temporal demand, and performance from the NASA RTLX questionnaire by Georgsson (2019) and one German version of the listening effort scaling question by Van Esch et al. (2013) on a continuous scale of 0–100. The questions from the NASA RTLX questionnaire were translated into German as already done by Schmutz et al. (2009) and Flägel et al. (2019), with ratings provided on a continuous scale from 0 to 100. The physical demand question was removed from the NASA RTLX questionnaire, as no specific physical activity was included in the experiment. This is also suggested by Helton et al. (2022) as physical demand may not be able to combine in a meaningful way with cognitive demands. For the performance rating of the NASA RTLX, the same continuous 0–100 scale (very low to very high) as for the other four questions from the NASA RTLX questionnaire was used to make subjects understand performance more intuitively (cf. Parkin et al., 2016; Said et al., 2020; Chen and Eickhoff, 2023). After answering a total of six questions after each trial, hence five from the NASA RTLX and one listening effort scaling question, the test supervisor again equipped the subjects with the HMD, headphones, and controller and continued the test. It was decided not to let the subjects answer the questionnaires in VR to give them a break from the virtual environment, as HTC also recommends it, the device manufacturer of the Vive Pro 2 HMD used in the experiments (cf. HTC Corporation, 2021).

After subjects completed the experiment with all nine trials, they were required to complete again the SSQ described above to get an insight into how the IVE usage impacted the participants' perceived amount of simulator sickness. Furthermore, participants were asked to complete the Igroup Presence Questionnaire (IPQ) by Schubert et al. (2001). It consists of 14 questions about general presence, spatial presence, participation, and experienced realism, each on a discrete scale from 0 to 6.

#### 3.4.4 Main part of the experiment

After the four-trial training session, subjects began the main part of the experiment. The task in each of the nine trials was the same as in the training session: to assign the stories to the correct speakers as quickly as possible, within a 2-min time limit. Unlike in Ahrens et al. (2019), participants in our study were not allowed to continue assigning stories once the 120 s playback ended, to avoid extending the experiment and to prevent a visual stagnation of the 360° video scene. The time limit was set to 120 s to prevent participants from overthinking or spending excessive time on speaker-story mappings, particularly during trials with a higher number of simultaneously active speakers. Additionally, the time limit was intended to simulate real-world time constraints. Since the recorded stories varied in length among the speakers, stories were repeated if they were under 120 s long. No algorithms were applied to alter the speech tempo in order to avoid possible side effects from slowing down the video in the 360° video experimental conditions. During the task, subjects were free to explore the virtual environment with three degrees of freedom, limited to head rotations only. It was decided not to allow six degrees of freedom, including body movements, as it would have added unnecessary complexity to the scenario and precluded direct comparisons with existing research in this domain, such as the study by Ahrens et al. (2019).

A mixed design was used for the conducted experiments, where the total number of simultaneously presented stories within each experimental condition was tested as a within-subject factor, and the three experimental conditions with their specific visual and acoustic instantiations, described in Section 3.5.1, were tested as between-subject factors. For each experiment, the nine trials were randomized in terms of the number of active speakers, while each participant experienced a unique set of nine trials. The trials differed in the total number of stories presented simultaneously, ranging from 2 to 10, each involving a different speaker. Because the levels of “total number of stories presented simultaneously” were deliberately varied, it is treated as a fixed factor in the analysis. A random drawing system ensured equally frequent usage of speakers and stories and avoided repetitions of individual speaker-story pairs. To prevent learning effects, the active speakers' locations were randomly selected on each trial. In Supplementary Figure S6, on the *x*-axis, the total number of stories presented simultaneously, and on the *y*-axis, the shortest circular distance in degrees between active speakers, with 95% confidence intervals (CI), is shown. It is visible that even though the locations of the active speakers were not controlled and randomly chosen, the distance between active speakers was constant between the three different experimental conditions. These measures ensured that each subject received a different combination of speakers and stories per trial, minimizing the influence of, e.g., learning effects on other dependent variables, e.g., task performance. Since 10 stories were recorded from each speaker in Fremerey et al. (2024), participants will hear the same story more than once during the nine trials, although it will be spoken by different speakers. This was necessary, as the effort required to record the dataset (cf. Fremerey et al., 2024) would have made it impractical to record 54 unique 2 m long stories from each speaker. To ensure an equal distribution of male and female speakers, differences in audio pitch were taken into account. This was carefully maintained for even numbers of speakers, while for odd numbers, the gender of the last speaker was chosen randomly. Additionally, for the first two experiments, the audiovisual recordings always matched the location of the talker, while in the third experiment, the audio was consistently paired with the CGI avatar modeled for the specific speaker.

For the first two tests, “360° binaural” and “360° diotic,” synchronous lip movements were given due to the video recording for active speakers, while non-active speakers remained silent but were still slightly moving. In the third test, “CGI binaural,” static CGI avatars without lip synchronization were presented. Hence, except for the talkers' gender, the listener had no visual indicator of who was speaking in the virtual room and had to rely solely on her or his auditory perception. Due to the high number of questions, the total test duration was about 70 min. To keep the overall testing time manageable and within acceptable limits, every participant experienced each of the nine trials once. During the experiments, other subject-related data, including head rotation data, Euler angles from the HMD, and timestamps, were recorded at the same frequency as the fixed frame rate of 90 fps in the Unity environment. Recording of this data began at the start of the trial and continued until its conclusion. Data were recorded at 90 Hz for the experimental conditions 360° diotic and 360° binaural and 45 Hz for the CGI binaural test. For the latter test, a lower recording frequency has been chosen to enable a smooth playout of the IVE.

### 3.5 Independent and dependent variables

As follows, the independent and dependent variables of the three different experiments, which will be evaluated in Section 4, are described.

#### 3.5.1 Independent variables

The independent variables of the three experiments were the visual and acoustic instantiations of the IVE used: 360° video with binaural audio for the first experiment, 360° video with diotic audio for the second experiment, and a CGI environment with binaural audio for the third experiment. Here, 360° with “binaural” can be considered as the reference with the best audiovisual match and intrinsic lip-sync. Another independent test variable for all three experiments was the number of stories presented simultaneously, which ranged from 2 to 10. One more independent variable is the distance between active speakers [°] and refers to the shortest circular distance in degrees between active speakers.

#### 3.5.2 Dependent variables

There were various dependent variables of the three conducted experiments with regard to measuring task performance. Based on the story-to-speaker mappings, we can calculate the percentage of correctly assigned stories. Furthermore, the total time required is another dependent variable related to task performance.

As already mentioned in Section 3.4.4, head rotation data in the form of Euler angles were recorded from each participant, from which a few dependent variables were derived. This includes the amount of time spent watching active speakers, which serves as one dependent variable. The total number of yaw degrees explored is also derived as a dependent variable from the head rotation data. This is computed as follows, with *i* representing the iterated yaw degree value and *n* as the last recorded yaw degree value of each recorded data sample:


$$\text{yaw\_explored}=\overset{n}{\underset{i=2}{\sum }}|\Delta y_{i}|$$

$$\Delta y_{i}=\{\begin{array}{ll} y_{i}-y_{i-1}, & \text{if }|y_{i}-y_{i-1}|\leq 180\\ y_{i}-y_{i-1}-360, & \text{if }(y_{i}-y_{i-1})>180\\ y_{i}-y_{i-1}+360, & \text{if }(y_{i}-y_{i-1})<-180 \end{array}$$

Another dependent variable derived from the head rotation data is the total number of yaw direction changes, which was computed as follows. At first, the differences were computed between each pair of two consecutive yaw values:


$$\Delta y_{i}=y_{i}-y_{i-1},\;\text{for}i=2,\ldots ,n$$

Then, the signs were computed with a threshold τ. It was defined as the so-called micromovements of the head (cf. Rossi et al., 2024) that would otherwise lead to an artificially high amount of yaw direction changes. Therefore, to compensate for the recording frequency of the head rotation data, τ was set to 0.1° for the experiments with the 360° experimental condition and to 0.2° for the experiment with the CGI experimental condition.


$${\text{sign}}_{i}=\{\begin{array}{ll} 1, & \text{if }\Delta y_{i}>\tau \\ -1, & \text{if }\Delta y_{i}<-\tau \\ 0, & \text{if }-\tau \leq \Delta y_{i}\leq \tau \end{array}$$

As a last step, initialize *count* = 0 and *prev*_*sign* = 0, while *count* is the total number of yaw direction changes per recorded data sample. For each *sign*_*i*_:

$$\begin{array}{l} \text{if}\left({\text{sign}}_{i}\neq 0\text{ and }{\text{sign}}_{i}\neq \text{prev\_sign}\right):\\ \;\text{count}=\text{count}+1,\text{prev\_sign}={\text{sign}}_{i} \end{array}$$

Another dependent variable, “Number of yaw direction changes per second,” refers to the total number of yaw direction changes divided by the total time needed per trial. The last dependent variable, “Deviation [°],” refers to the amount of degrees that subjects have deviated if they have recognized the right story *per se* but have assigned it to the wrong speaker.

Further dependent variables were derived from the questionnaires administered to the subjects. That includes the five single dimensions of the NASA RTLX questionnaire mentioned in Section 3.4.3, hence NASA RTLX effort, frustration, mental demand, performance and temporal demand. Further, from the recorded NASA RTLX values, one unweighted score, called the total NASA RTLX mental workload score, was calculated by averaging the raw data from the five factors. Another dependent variable, listening effort, was derived from the listening effort scaling question mentioned in Section 3.4.3. Further dependent variables were derived from the SSQ mentioned in Section 3.4.3, including nausea (N), oculomotor (O), disorientation (D) and the total score (TS), which is computed from the three single scores. Other dependent variables are derived from the IPQ mentioned in Section 3.4.3, hence general presence (G1), spatial presence (SP), involvement (INV), and experienced realism (REAL).

### 3.6 Dataset

All recorded data from the three experiments are made publicly available.^3^ This includes speaker-story mappings, head rotation data, NASA RTLX and effort scaling questionnaires, and SSQ, IPQ, and Weinstein questionnaire results.

### 3.7 Technical setup and equipment

The PC used for the subjective tests was running Windows 11 and equipped with an Intel Core i7-13700K with 64 GB RAM, a Samsung SSD 980 Pro 2 TB, and an NVIDIA RTX 4090 graphics card. Unity version 2019.4.17f1 was used to run all IVEs. An HTC Vive Pro 2 was connected to the PC, together with an RME Fireface UCX II sound card and Sennheiser HD 650 headphones. As described in Fremerey et al. (2024), the 360° videos were encoded using the High Efficiency Video Coding (HEVC) implementation of FFmpeg 6.0 (libx265) with a Constant Rate Factor (CRF) of 1 and chroma subsampling of 4:2:0, resulting in a visually lossless encoding while supporting hardware-accelerated decoding for smooth playback.

The audio post-processing is described in more detail in Fremerey et al. (2024). An FFT filter from Adobe Audition 1.5 with an FFT size of 1,892 was used to filter frequencies above 13–14 kHz, mitigating ambient noises from, e.g., lamps and cameras. The denoising noise reduction plugin from Adobe Audition was only necessary for 2–3 speakers; further audio peaks have been smoothed out. Normalization was applied according to the EBU R128 standard (EBU Recommendation, 2023) to all recorded audio signals.

To play back one speaker with an average sound pressure level of 60 dB(A), several measurements have been performed. Measurements were carried out with the GRAS KEMAR dummy head with torso, including pinna models GRAS KB0060 and KB0061, 1/2″ microphone capsules type 40AO, microphone preamplifier type 26CS, and IEPE conditioning modules M32. The dummy head was placed 1.3 m from a Genelec 8341A loudspeaker, representing the distance from the listener, placed in the middle of the circle of chairs, to one speaker in all three experiments. The loudspeaker was connected to a PC that played pink noise using VAUnity, the auralization engine used for both IVEs, 360° video, and CGI. To aim for a later sound pressure level of 60 dB(A) in the headphones, as a first step, the volume of the loudspeaker was adjusted in the sound card so that a sound pressure level of 60 dB(A) was measured at the same height of the artificial head using the NTI XL2 audio analyzer. At the same time, the input levels of the artificial head were measured with the microphone capsules integrated in the dummy head. Afterwards, headphones were placed on the dummy head. As a second step, pink noise was again played back via VAUnity, whereby the volume on the sound card was set so that the input level measured by the microphones of the dummy head was reached. These measurements and the respective settings of the sound card were performed for both diotic and binaural audio. For verification purposes, the measurement was done again with one speaker as the audio signal instead of pink noise to ensure that an average sound pressure level of 60 dB(A) was achieved for a speaker at a recording time of 10 s.

## 4 Results

For each trial, the speaker-story mappings, head-rotation values in Euler angles (pitch, yaw, and roll, respectively), and results for the NASA RTLX and effort scaling questionnaire have been recorded. Furthermore, the total time passed until the trial was finished by the subject or, if the subject had exceeded the maximum allowed time of 120 s, this respective maximum was recorded. In addition, the Weinstein noise sensitivity score, the SSQ scores before and after the test, and the IPQ scores per subject were obtained and calculated. As indicated in Supplementary Section 1.1 and Supplementary Figure S4, noise sensitivity scores are consistent across the three participant groups of the conducted experiments, which is why we decided not to investigate this further.

To investigate whether the independent variables had an influence on the dependent variables for the different auditory and visual conditions of the tests, an Aligned Rank Transform (ART) (Wobbrock et al., 2011) has been performed using R v4.4.2 with the ARTool package v0.11.1 (Kay et al., 2021), followed by a nonparametric analysis of variance (ANOVA) applied to the transformed data according to Wobbrock et al. (2011) and subsequent Bonferroni-corrected contrast tests. As Shapiro-Wilk tests revealed that the data were not normally distributed and Levene's test indicated a violation of homoscedasticity, it was decided not to perform a mixed analysis of variance. Since the recorded data contains very few outliers and not for every total story detected by boxplot outlier detection, as shown in Supplementary Figure S1, it was decided to analyze all subjects as they are and not to exclude any.

### 4.1 Influence of total number of stories and experimental condition on percentage of correctly assigned stories

**Figure 3:**
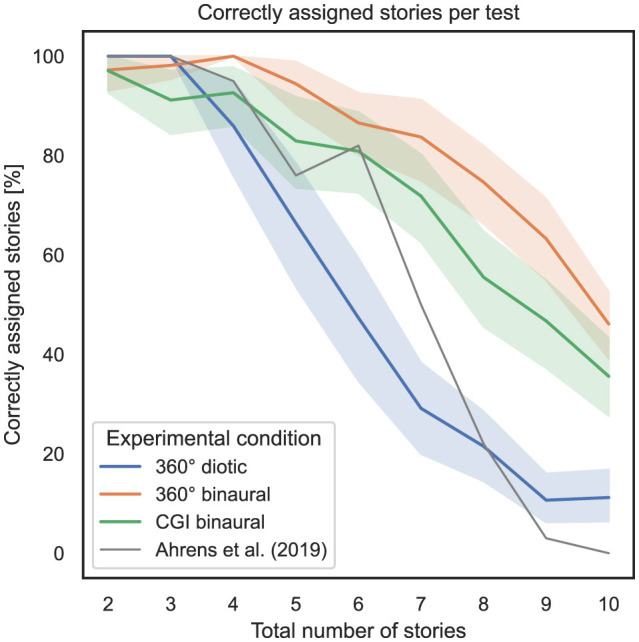
Total number of stories (independent variable) vs. percentage of correctly assigned stories per test (dependent variable) across the three different experimental conditions (cf. RQ1, RQ2 in Section 1).

To answer RQ1 and RQ2 stated in Section 1, the influence of the total number of stories presented simultaneously and the experimental condition (i.e., specific experiment) on the percentage of correctly assigned stories as a measure of task performance is analyzed. Figure 3 shows, on the *x*-axis, the total number of stories presented simultaneously, and on the *y*-axis, the percentage of correctly assigned stories, with 95% confidence intervals (CI). The gray line represents the mean percentage of correctly assigned stories per total number of stories simultaneously presented for the study conducted by Ahrens et al. (2019).

To compute the ART model, the number of correctly assigned stories per trial was defined as the dependent variable, the total number of simultaneously presented stories as the within-subject variable, and the experimental condition as the between-subject variable. Subsequently, a nonparametric mixed ANOVA has been applied to the transformed data, while the detailed results are stated in Supplementary Section 1.2. Subsequent Bonferroni-corrected contrast tests revealed that the experimental condition used in general had a significant impact on the percentage of correctly assigned stories; therefore, between 360° diotic and 360° binaural (*p* = 1.03*10^−13^), between 360° diotic and CGI binaural (*p* = 8.16*10^−7^) and between 360° binaural and CGI binaural (*p* = 3.9*10^−4^). Table 1 presents the results of Bonferroni-corrected contrast tests, with the percentage of correctly assigned stories as the dependent variable.

**Table 1:** Bonferroni-corrected contrast tests from ART model, dependent variable: percentage of correctly assigned stories.

**Contrast**	**#S**	**A**	**B**	***p*-corr**
#S * T	5	360° diotic	360° binaural	1.08·10^−6^
#S * T	6	360° diotic	360° binaural	5.98·10^−11^
#S * T	6	360° diotic	CGI binaural	1.43·10^−7^
#S * T	7	360° diotic	360° binaural	1.21·10^−17^
#S * T	7	360° diotic	CGI binaural	9.66·10^−9^
#S * T	7	360° binaural	CGI binaural	0.045
#S * T	8	360° diotic	360° binaural	2.59·10^−13^
#S * T	8	360° diotic	CGI binaural	0.0001
#S * T	8	360° binaural	CGI binaural	0.009
#S * T	9	360° diotic	360° binaural	9.02·10^−10^
#S * T	9	360° diotic	CGI binaural	0.0003
#S * T	10	360° diotic	360° binaural	0.0004

Only significant effects reported.

#S * T refers to the total number of stories presented simultaneously * the experimental condition specified in “A” and “B” (cf. RQ1, RQ2 in Section 1).

To examine the correspondence with regard to task performance, measured by the percentage of correctly assigned stories, between the experiments in this study and the study by Ahrens et al. (2019), Pearson correlation coefficients (PCC) were calculated for each experimental condition, which is a key aspect of RQ1. Between the 360° diotic and the test from Ahrens et al. (2019), a PCC of 0.81, *p* = 1.79*10^−52^ was calculated. Between the 360° binaural and the test from Ahrens et al. (2019), a PCC of 0.63, *p* = 1.2*10^−37^ was computed. Between the CGI binaural and the test from Ahrens et al. (2019), a PCC of 0.65, *p* = 3.47*10^−38^ was calculated.

In summary, regarding RQ1, it can be stated that hypothesis H1 is partially falsified concerning the correspondence of the percentage of correctly assigned stories. A strong correspondence to the results of the study by Ahrens et al. (2019) is evident only for the 360° diotic test. In contrast, the 360° binaural and CGI binaural experimental conditions only show a moderate correspondence with the findings of Ahrens et al. (2019). Regarding RQ2, hypothesis H2 can be confirmed, particularly in relation to the assertion that an increasing total number of stories presented simultaneously results in decreasing task performance. This effect is more visible in diotic compared to binaural audio conditions and in the CGI representation compared to 360° video.

### 4.2 Influence of total number of stories and experimental condition on task completion time

**Figure 4:**
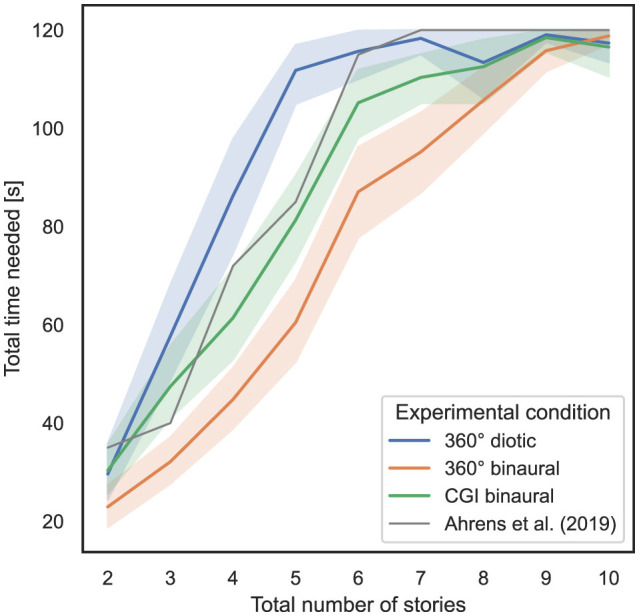
Total number of stories (independent variable) vs. total time needed per test (dependent variable) across the three different experimental conditions (cf. RQ1, RQ2 in Section 1).

In order to answer RQ1 and RQ2 stated in Section 1, the influence of the total number of stories presented simultaneously and the experimental condition used on task completion time as a measure of task performance is analyzed. In Figure 4, the *x*-axis represents the total number of stories presented simultaneously, while the *y*-axis shows the total time required for all responses, including incorrect ones, with 95% CIs. The gray line represents the mean time required per total number of stories narrated simultaneously for the listeners of the experiment conducted by Ahrens et al. (2019). It should be noted that in that experiment, the subjects were still able to give their rating after the stories were played for 120 s, which was not possible in the experiments conducted in the present study. The last four values, therefore, for a total number of stories greater than seven, the values in the original study were greater than 120 s and clipped to 120 s for this presentation.

To compute the ART model, the total time needed per trial was defined as a dependent variable, the total number of stories simultaneously presented was defined as a within-subject variable, and the experimental condition was defined as a between-subject variable. Subsequently, a nonparametric mixed ANOVA has been applied to the transformed data, while the detailed results are stated in Supplementary Section 1.3. Bonferroni-corrected contrast tests quantitatively prove that the experimental condition used in general had a significant impact on the total time needed; therefore, between 360° diotic and 360° binaural (*p* = 3.4*10^−13^), between 360° diotic and CGI binaural (*p* = 2.58*10^−5^) and between 360° binaural and CGI binaural (*p* = 3.37*10^−5^). Table 2 presents the results of Bonferroni-corrected contrast tests, with the total time needed as the dependent variable.

**Table 2:** Results of Bonferroni-corrected contrast tests applied to computed ART model, dependent variable: total time needed.

**Contrast**	**#S**	**A**	**B**	***p*-corr**
#S * T	3	360° diotic	360° binaural	0.0006
#S * T	4	360° diotic	360° binaural	4.97*10^−11^
#S * T	4	360° diotic	CGI binaural	0.0001
#S * T	5	360° diotic	360° binaural	5.85*10^−18^
#S * T	5	360° diotic	CGI binaural	2.79*10^−7^
#S * T	5	360° binaural	CGI binaural	0.004
#S * T	6	360° diotic	360° binaural	1.3*10^−7^
#S * T	6	360° binaural	CGI binaural	0.009
#S * T	7	360° diotic	360° binaural	3.98*10^−7^
#S * T	7	360° binaural	CGI binaural	0.006

Only significant effects reported.

#S * T refers to the total number of stories presented simultaneously * the experimental condition specified in “A” and “B” (cf. RQ1, RQ2 in Section 1).

To examine the correspondence with regard to task performance, measured by the total time needed, between the experiments in this study and the study by Ahrens et al. (2019), Pearson correlation coefficients (PCC) were calculated for each experimental condition, which is a key aspect of RQ1. Between the 360° diotic and the test from Ahrens et al. (2019), a PCC of 0.83, *p* = 3.77*10^−58^ was calculated. Between the 360° binaural and the test from Ahrens et al. (2019), a PCC of 0.84, *p* = 1.81*10^−86^ was computed. Between the CGI binaural and the test from Ahrens et al. (2019), a PCC of 0.84, *p* = 6.54*10^−82^ was calculated.

In summary, regarding RQ1, hypothesis H1 is confirmed with respect to the correspondence with regard to total time needed. A strong correspondence with the findings of Ahrens et al. (2019) is evident across all experimental conditions. For RQ2, hypothesis H2 is also confirmed, particularly supporting the claim that an increasing total number of stories presented simultaneously leads to an increase in total time needed, which subsequently reduces task performance. This effect is more visible in diotic compared to binaural audio conditions and in the CGI representation compared to 360° video.

### 4.3 Influence of total number of stories and experimental condition on mental load

In order to answer RQ2 stated in Section 1, which aims to investigate how the total number of simultaneously presented stories, the audio condition (binaural vs. diotic), and the visual IVE representation (360° video vs. CGI) impact the task performance of subjects, the influence of the total number of stories presented simultaneously and the experimental condition on the perceived mental load of subjects is analyzed. After each trial, subjects were required to complete the NASA RTLX questionnaire, which consists of five questions on perceived effort, frustration, mental demand, performance, and temporal demand. Additionally, a scaling effort question was asked after each trial. The NASA RTLX performance rating was inverted before evaluation as the scale was inverted, as stated in Section 3.

**Figure 5:**
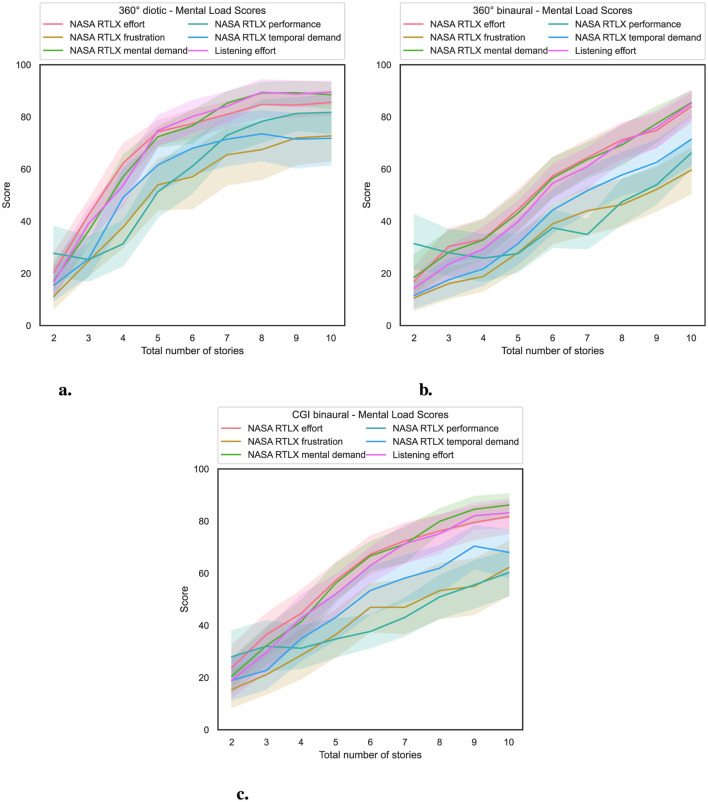
Total number of stories (independent variable) vs. mental load score (dependent variable) for the three experimental conditions (cf. RQ2 in Section 1). **(a)** 360° diotic. **(b)** 360° binaural. **(c)** CGI binaural.

To investigate RQ2, in Figure 5, on the *x*-axis, the total number of stories presented simultaneously is shown, and on the *y*-axis, the mental load score of the specific dimension from the NASA RTLX or listening effort questionnaire.

An increasing total number of simultaneously presented stories results in a corresponding increase in mental load.

For statistical analysis, an ART model was computed, with the respective score defined as the dependent variable, the total number of stories presented simultaneously as the within-subject variable, and the experimental condition as the between-subject variable. That was followed by a non-parametric mixed ANOVA, which was applied to the transformed data. The results of the nonparametric mixed ANOVAs are shown in Supplementary Table S1 and show that the interaction effect between the specific test and the score of the respective question used is statistically significant. This indicates that the effect of the experimental condition on the specific score varies. Hence, the difference between experimental conditions is not uniform at all story levels across all dimensions of NASA RTLX and listening effort. In addition, there was a notable effect of the total number of stories presented simultaneously on the scores for the respective questions used, again across all dimensions of the NASA RTLX and listening effort. Furthermore, across all dimensions of NASA RTLX and listening effort, the experimental condition had a significant effect on the scores of the respective questions.

The results of the Bonferroni-corrected contrast tests are shown in Supplementary Table S2. The results reveal that the experimental condition used had a significant impact on NASA RTLX and listening effort scores, depending on the number of stories presented simultaneously.

In summary, regarding RQ2, hypothesis H2 is confirmed, as the total number of stories presented simultaneously increases, resulting in a corresponding increase in mental load for participants, as shown in Figure 5. Significant effects were observed between the 360° diotic and binaural experimental conditions, as well as between the 360° diotic and CGI binaural experimental conditions, particularly when the total number of stories presented simultaneously exceeded four. However, no significant differences in mental load were found between the 360° binaural and the CGI binaural experimental conditions.

### 4.4 Influence of total number of stories and experimental condition on proportion of time spent watching active speakers

**Figure 6:**
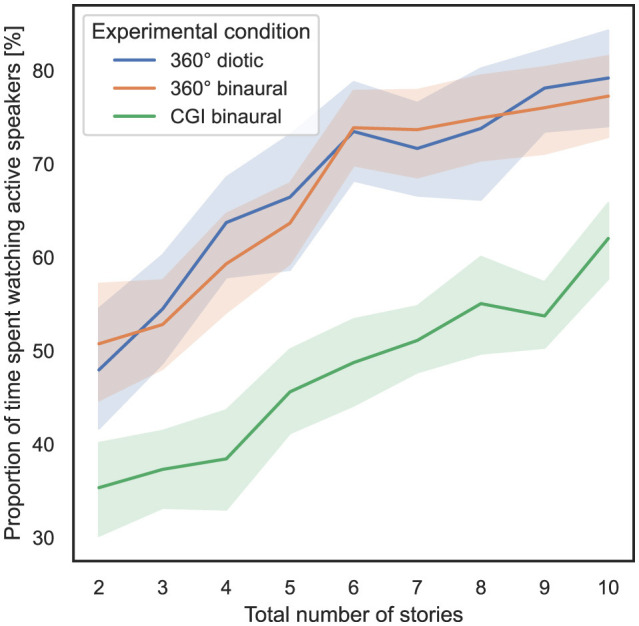
Total number of stories (independent variable) vs. proportion of time spent watching active speakers per test (dependent variable) across the three different experimental conditions (cf. RQ3 in Section 1).

In order to answer RQ3 stated in Section 1, the influence of the total number of stories presented simultaneously and the experimental condition on the proportion of time spent watching active speakers, as defined in Section 3.5.2, is analyzed. In Figure 6, the total number of stories presented simultaneously is shown on the *x*-axis, and the proportion of time spent watching active speakers, with 95% CIs, is shown on the *y*-axis. For the three tests conducted, always 20 speakers were included in the IVE, and hence the scene was divided into 18° wide yaw angle ranges. The head rotation yaw values of the subjects were mapped to the specific ranges of active speakers, hence speakers who narrate a story in the specific trial.

To compute the ART model, the proportion of time spent watching active speakers is defined as the dependent variable, the total number of stories presented simultaneously is defined as the within-subject variable, and the experimental condition is defined as the between-subject variable. Subsequently, a non-parametric mixed ANOVA has been applied to the transformed data, while the detailed results are stated in Supplementary Section 1.4. Bonferroni-corrected contrast tests show that the experimental condition used had a significant impact on the proportion of time spent watching active speakers between the 360° diotic and CGI binaural (*p* = 5.88*10^−15^) and between the 360° binaural and CGI binaural experimental conditions (*p* = 5.1*10^−16^). It did not have a significant impact between the 360° diotic and the 360° binaural experimental conditions (*p* = 0.685). Table 3 presents the results of Bonferroni-corrected contrast tests, with the total time needed as the dependent variable.

**Table 3:** Results of Bonferroni-corrected contrast tests applied to computed ART model, dependent variable: proportion of time spent watching active speakers.

**Contrast**	**#S**	**A**	**B**	***p*-corr**
#S * T	2	360° binaural	CGI binaural	0.004
#S * T	3	360° diotic	CGI binaural	0.0006
#S * T	3	360° binaural	CGI binaural	0.001
#S * T	4	360° diotic	CGI binaural	2.14*10^−8^
#S * T	4	360° binaural	CGI binaural	3.38*10^−7^
#S * T	5	360° diotic	CGI binaural	8.55*10^−8^
#S * T	5	360° binaural	CGI binaural	4.03*10^−6^
#S * T	6	360° diotic	CGI binaural	5.15*10^−10^
#S * T	6	360° binaural	CGI binaural	1.45*10^−12^
#S * T	7	360° diotic	CGI binaural	1.49*10^−7^
#S * T	7	360° binaural	CGI binaural	6.48*10^−11^
#S * T	8	360° diotic	CGI binaural	1.3*10^−5^
#S * T	8	360° binaural	CGI binaural	1.89*10^−7^
#S * T	9	360° diotic	CGI binaural	2.73*10^−10^
#S * T	9	360° binaural	CGI binaural	2.96*10^−10^
#S * T	10	360° diotic	CGI binaural	0.0002
#S * T	10	360° binaural	CGI binaural	0.0002

Only significant effects were reported.

#S * T refers to the total number of stories presented simultaneously * the experimental condition specified in “A” and “B” (cf. RQ3 in Section 1).

In summary, regarding RQ3, hypothesis H3 can be partially confirmed, as the proportion of time spent watching active speakers significantly differs between the 360° diotic and CGI binaural experimental conditions and between the 360° binaural and CGI binaural experimental conditions. However, no significant difference is observed between the 360° diotic and binaural experimental conditions.

### 4.5 Influence of total number of stories and experimental condition on total yaw degrees explored

**Figure 7:**
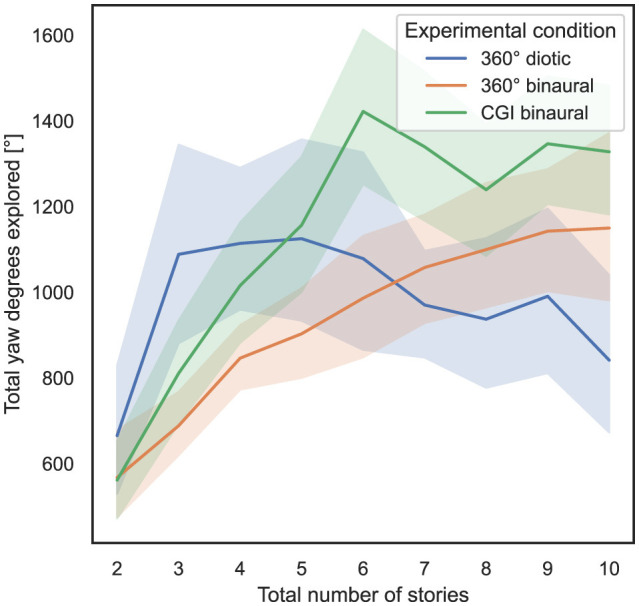
Total number of stories (independent variable) vs. total yaw degrees explored (dependent variable) across the three different experimental conditions (cf. RQ3 in Section 1).

In order to answer RQ3 stated in Section 1, the influence of the total number of stories presented simultaneously and the experimental condition on the total amount of yaw degrees explored, as defined in Section 3.5.2, is analyzed. In Figure 7, the total number of stories presented simultaneously is shown on the *x*-axis, and on the *y*-axis, the total yaw degrees explored, with 95% CIs.

As a result of the experiments, subjects primarily turned their heads and did not move them up or down more than necessary to look at the controller. Hence, in the following behavioral analysis, the focus is placed on yaw values.

To compute the ART model, the total yaw degrees explored were defined as the dependent variable, the total number of stories presented simultaneously was defined as the within-subject variable, and the experimental condition was defined as the between-subject variable. Subsequently, a nonparametric mixed ANOVA has been applied to the transformed data, while the detailed results are stated in Supplementary Section 1.5. Bonferroni-corrected contrast tests revealed that the experimental condition used had a significant impact on the total yaw degrees explored between the 360° diotic and the CGI binaural test (*p* = 0.012) and between the 360° binaural and CGI binaural experimental conditions (*p* = 0.004), but not between the 360° diotic and the 360° binaural experimental conditions (*p* = 0.867). Table 4 presents the results of Bonferroni-corrected contrast tests, with the total yaw degrees explored as the dependent variable.

**Table 4:** Results of Bonferroni-corrected contrast tests applied to computed ART model, dependent variable: total yaw degrees explored.

**Contrast**	**#S**	**A**	**B**	***p*-corr**
#S * T	3	360° diotic	360° binaural	0.007
#S * T	6	360° diotic	CGI binaural	0.027
#S * T	6	360° binaural	CGI binaural	0.001
#S * T	9	360° diotic	CGI binaural	0.016
#S * T	10	360° diotic	CGI binaural	4.45*10^−5^

Only significant effects were reported.

#S * T refers to the total number of stories presented simultaneously * the experimental condition specified in “A” and “B” (cf. RQ3 in Section 1).

In summary, regarding RQ3, hypothesis H3 can be partially falsified in relation to the total yaw degrees explored, as no significant differences were observed between most of the experimental conditions.

### 4.6 Influence of total number of stories and experimental condition on yaw direction changes

**Figure 8:**
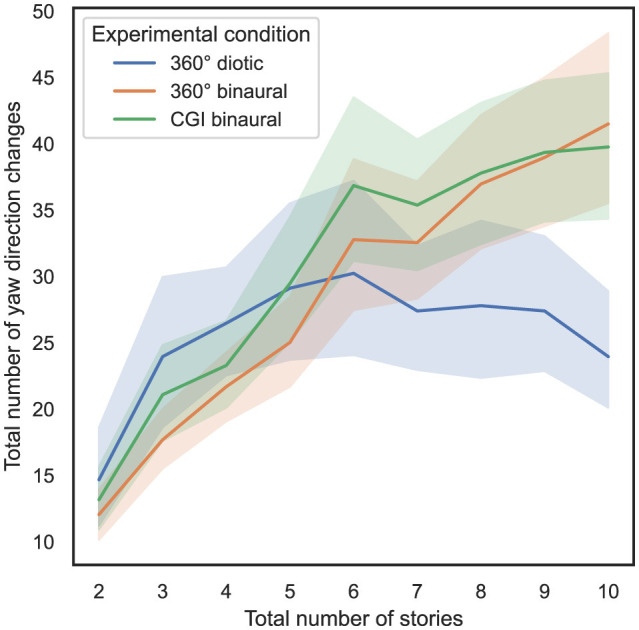
Total number of stories (independent variable) vs. total number of yaw direction changes (dependent variable) across the three different experimental conditions (cf. RQ3 in Section 1).

In order to answer RQ3 stated in Section 1, the influence of the total number of stories presented simultaneously and the experimental condition on the total amount of yaw direction changes, as defined in Section 3.5.2, is analyzed. In Figure 8, on the *x*-axis, the total number of stories presented simultaneously is plotted vs. the total number of yaw direction changes on the *y*-axis, with 95% CIs.

To compute the ART model, the total number of yaw direction changes was defined as dependent variable, the total number of stories presented simultaneously was defined as within-subject variable, and the experimental condition was defined as between-subject variable. Subsequently, a nonparametric mixed ANOVA has been applied to the transformed data, while the detailed results are stated in Supplementary Section 1.6. Bonferroni-corrected contrast tests reveal that the experimental condition used only had a significant impact on the total number of yaw direction changes between the 360° diotic and the CGI binaural test (*p* < 0.05). Table 5 presents the results of Bonferroni-corrected contrast tests, with the total number of yaw direction changes as the dependent variable.

**Table 5:** Results of Bonferroni-corrected contrast tests applied to computed ART model, dependent variable: total number of yaw direction changes.

**Contrast**	**#S**	**A**	**B**	***p*-corr**
#S * T	9	360° diotic	360° binaural	0.037
#S * T	9	360° diotic	CGI binaural	0.023
#S * T	10	360° diotic	360° binaural	0.0004
#S * T	10	360° diotic	CGI binaural	0.0004

Only significant effects reported.

#S * T refers to the total number of stories presented simultaneously * the experimental condition specified in “A” and “B” (cf. RQ3 in Section 1).

**Figure 9:**
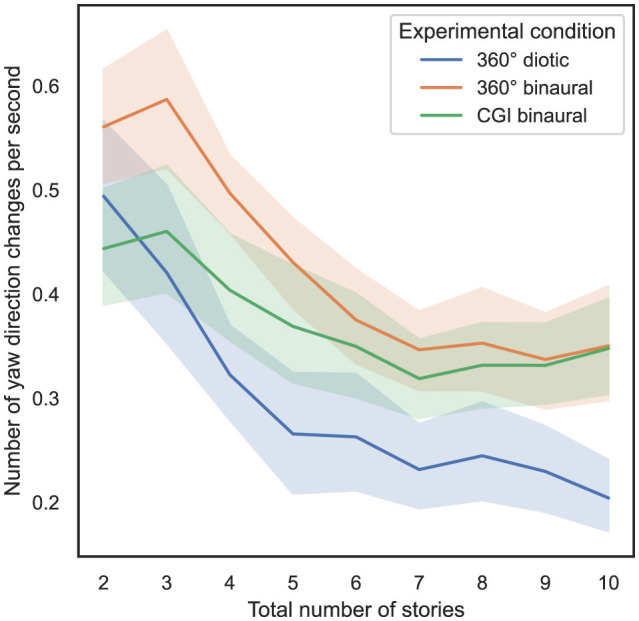
Total number of stories (independent variable) vs. number of yaw direction changes per second (dependent variable) across the three different experimental conditions (cf. RQ3 in Section 1).

Additionally, in Figure 9 another variant of this analysis is shown, while on the *y*-axis the number of yaw direction changes per second is shown, with 95% CIs.

From Figure 9, it can be observed that as the number of stories presented simultaneously increases, the number of yaw direction changes per second decreases. To compute the ART model, the number of yaw direction changes per second was defined as a dependent variable, the total number of stories presented simultaneously was defined as a within-subject variable, and the experimental condition was defined as a between-subject variable. Subsequently, a nonparametric mixed ANOVA has been applied to the transformed data, while the detailed results are stated in Supplementary Section 1.6. Bonferroni-corrected contrast tests reveal that the experimental condition used had a significant impact on the number of yaw direction changes per second between all different tests (*p* < 0.05). Table 6 presents the results of Bonferroni-corrected contrast tests, with the number of yaw direction changes per second as the dependent variable.

**Table 6:** Results of Bonferroni-corrected contrast tests applied to computed ART model, dependent variable: number of yaw direction changes per second.

**Contrast**	**#S**	**A**	**B**	***p*-corr**
#S * T	3	360° diotic	360° binaural	0.029
#S * T	4	360° diotic	360° binaural	0.00006
#S * T	5	360° diotic	360° binaural	0.00005
#S * T	6	360° diotic	360° binaural	0.006
#S * T	7	360° diotic	360° binaural	0.008
#S * T	8	360° diotic	360° binaural	0.028
#S * T	9	360° diotic	360° binaural	0.02
#S * T	9	360° diotic	CGI binaural	0.03
#S * T	10	360° diotic	360° binaural	0.0008
#S * T	10	360° diotic	CGI binaural	0.0008

Only significant effects were reported.

#S * T refers to the total number of stories presented simultaneously * the experimental condition specified in “A” and “B” (cf. RQ3 in Section 1).

In summary, regarding RQ3, hypothesis H3 can be partially falsified in relation to the total number of yaw direction changes, as no significant differences were observed between most of the experimental conditions. Regarding the number of yaw direction changes per second, significant differences can mainly be observed between the 360° diotic and binaural experimental conditions across almost all levels of total stories presented simultaneously.

### 4.7 Influence of experimental condition and time of measurement on simulator sickness

**Figure 10:**
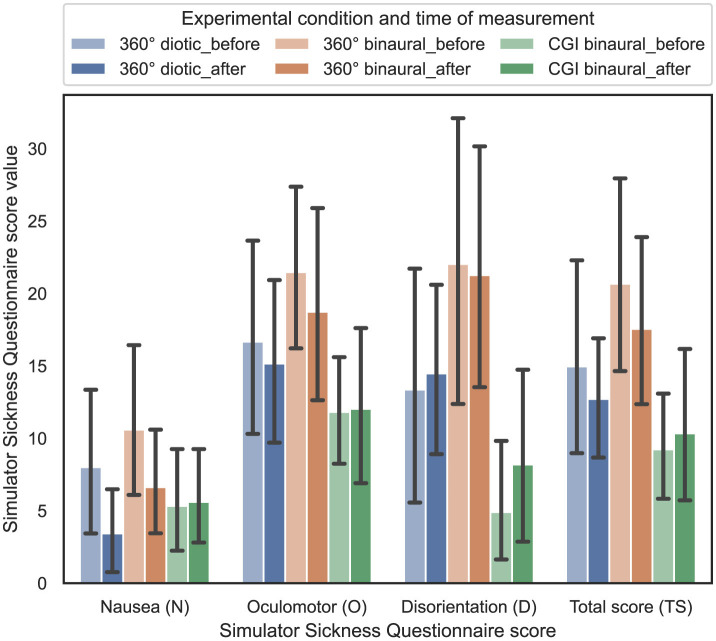
Simulator Sickness Questionnaire score values (dependent variable) vs. experimental condition and time of measurement (independent variable), that is, before or after the test across the three different experimental conditions (cf. RQ4 in Section 1).

In order to answer RQ4 stated in Section 1, the influence of the experimental condition and time of measurement on the perceived amount of simulator sickness is analyzed. Based on Kennedy et al. (1993), three different scores of the SSQ were calculated from the questions, resulting in nausea (N), oculomotor (O), and disorientation (D). From these three single scores, the total score (TS) was computed. In Figure 10, the resulting SSQ scores before and after each test are shown with 95% CIs.

For statistical analysis, an ART model has been computed for all four different SSQ scores. In contrast, the respective SSQ score was defined as the dependent variable, while the time of measurement—before or after the test—was defined as the within-subject variable. Afterwards, nonparametric one-way ANOVAs were calculated for the four different SSQ scores. The results revealed that the effect of the time of measurement is statistically significant for the nausea score [*F*_(1, 165)_ = 4.27, *p* = 0.04]. Further, the results revealed that the effect of the used experimental condition is statistically significant for the oculomotor score [*F*_(2, 146)_ = 4.49, *p* = 0.013], the disorientation score [*F*_(2, 146)_ = 8.41, *p* = 0.0003], and the total score [*F*_(2, 146)_ = 6.88, *p* = 0.001]. However, subsequent Bonferroni-corrected contrast tests indicate that the effect of the combination of experimental condition and time of measurement on each of the SSQ scores was not significant (*p*>0.05).

In summary, regarding RQ4, hypothesis H4 is confirmed, as simulator sickness levels (before/after the experiment) do not differ significantly between the visual IVE representations or audio conditions.

### 4.8 Influence of experimental condition on presence

**Figure 11:**
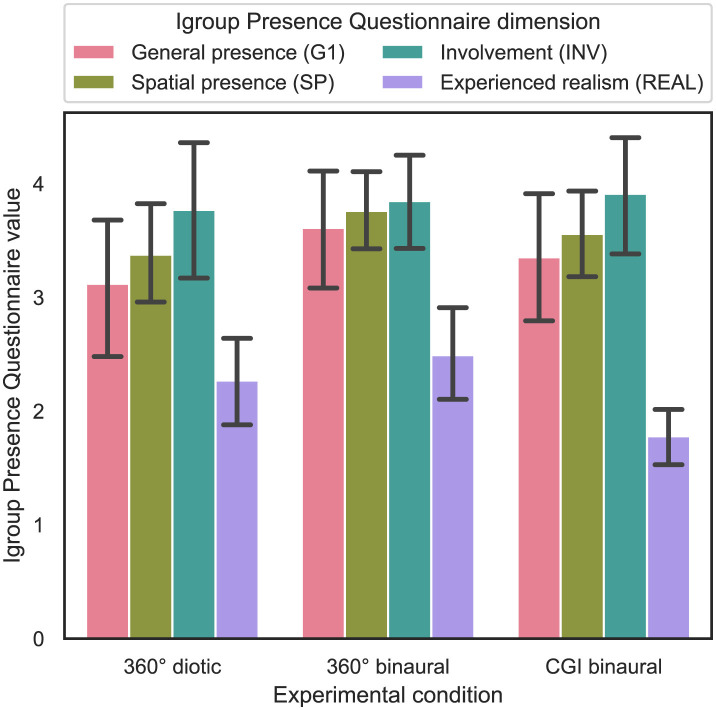
Experimental condition (independent variable) vs. Igroup Presence Questionnaire dimension values (dependent variable) across the three different experimental conditions (cf. RQ4 in Section 1).

In order to answer RQ4 stated in Section 1, the influence of the experimental condition on the perceived amount of presence is analyzed. In all three subjective tests, participants were asked to complete the IPQ once after the experiment. Based on Schubert et al. (2001), four different dimensions of the IPQ were calculated from the questions, namely general presence (G1), spatial presence (SP), participation (INV) and experienced realism (REAL). In Figure 11, the resulting IPQ scores are shown with 95% CIs.

For statistical analysis, an ART model was computed for all four different IPQ dimensions, with the IPQ score as the dependent variable and the experimental condition as a between-subject variable. Subsequently, a nonparametric one-way ANOVA was calculated for the four different IPQ dimensions. The results show that only the effect of the experimental condition on the REAL dimension is statistically significant [*F*_(2, 92)_ = 4.43, *p* = 0.015]. Subsequent Bonferroni-corrected contrast tests indicate that the effect of the experimental condition on the REAL dimension is only statistically significant between the 360° binaural and the CGI binaural experimental conditions, also reflected by the means (*p* = 0.015, 2.5 vs. 1.78). For the other dimensions of the IPQ, no statistically significant effects could be found.

In summary, regarding RQ4, hypothesis H4 is falsified, as presence levels do not differ significantly between the visual IVE representations or audio conditions, except for the REAL dimension, where a significant difference was observed between the 360° binaural and CGI binaural experimental conditions.

## 5 Discussion

This study proposes a psychometric method to investigate audiovisual interaction effects by measuring cognitive performance in three different audiovisual experimental conditions with good speech intelligibility. To do so, either 360° video or CGI was used for the visual representation of speakers and two different acoustic conditions, diotic and binaural audio. To this end, three different experimental conditions were investigated between subjects in three experiments: 360° diotic, 360° binaural, and CGI binaural. In all three experiments, subjects had the same task of correctly assigning the story to the respective speaker, while up to 10 stories were played simultaneously, and visually, all 20 possible speakers were always shown. Understanding cognitive performance in these contexts, even under extreme conditions with 10 concurrent speakers, mainly facilitates a deeper understanding of audiovisual interplay and the limitations of attentional mechanisms using a realistic and controlled IVE setting. The observed differences between CGI and 360° video conditions, particularly regarding the absence of lip-sync in the CGI scenario, underline the role of audiovisual interactions in evaluating audiovisual scene analysis performance. Although the hypotheses did not explicitly focus on audiovisual interaction, the results clearly demonstrate that subtle audiovisual cues, such as synchronized speech movements, significantly impact cognitive load and task performance. This study quantifies the scalability of audiovisual performance and cognitive load, leading to an understanding of audiovisual aspects that extends beyond simplified experimental scenarios often used in previous research. It also provides a valuable approach for improving real-world classroom acoustics in the future, for example, by using the integrated Virtual Acoustics naturalization software to simulate specific classroom acoustic settings. The answers to the four research questions raised at the beginning of this study in Section 1 and whether the four hypotheses also stated could be verified or falsified will be discussed as follows.

### 5.1 Correspondence of task performance with a previous study by Ahrens et al.

With RQ1, cf. Section 1, it was aimed to investigate how the task performance correlates with the results of Ahrens et al. (2019) for different levels of the total number of stories presented simultaneously across the two visual IVE representations (360° video vs. CGI) and audio conditions (binaural vs. diotic). In H1, cf. Section 1, it was hypothesized that task performance in both visual IVE representations (360° video and CGI) shows a strong correspondence with the performance observed in the study by Ahrens et al. (2019), regardless of whether the audio condition is binaural or diotic.

It should be mentioned that the results of the previous study by Ahrens et al. (2019) cannot be directly compared with the three experiments carried out in this study, as some parts of the original experimental design have been changed. This relates, for example, to the general visual design of the scene, with a visually more detailed approach with 20 different persons sitting in a circle of chairs around the listener, followed here. In the setup of Ahrens et al. (2019), 21 speaker locations were semi-circularly arranged around the listener between −90° and 90°, in 30° steps with three possible distances between the listener and the sources. In addition, the visual representations used in this study were designed to appear more realistic compared to the approach of Ahrens et al. (2019), which employed a rather simple visual scene with semi-transparent avatar silhouettes.

Furthermore, the approach of Ahrens et al. (2019) showed a clock in the back, indicating the remaining time in the current scene, while in the approach of this study, this was not done to avoid putting the test subjects under too much time pressure. Unlike in the study by Ahrens et al. (2019), in which subjects could complete their assignments after playing the stories for 120 s, this was not possible in the experiments presented during this study.

Nevertheless, the holistic task design in the study by Ahrens et al. (2019) remains pretty comparable to the design of the three experiments conducted in this study, as in both participants listened to between two and 10 stories at the same time, while the topics of the stories were the same.

As stated in Section 4.1 and indicated in Figure 3, the results of the study by Ahrens et al. (2019) are moderately correlated to the results of the 360° and CGI binaural experimental conditions carried out in this study and highly correlated to the results of the 360° diotic study, with respect to the percentage of stories that could be correctly assigned. This partially falsifies hypothesis H1, where it was stated that task performance shows a strong correspondence to the results of the study by Ahrens et al. (2019) for both visual IVE representations (360° video and CGI), which holds true for the 360° diotic study. The fact that, especially for a higher total number of stories (>6), the 360° and CGI binaural experimental conditions show a higher mean of the percentage of correctly assigned stories, as in the study by Ahrens et al. (2019), shows the positive impact of a more sophisticated audiovisual representation and hence medial representation of the virtual environment on task performance and the need for further research. Interestingly, as stated in Section 4.2 and indicated in Figure 4, the results of the study by Ahrens et al. (2019) are highly correlated to the results of all the three different experimental conditions carried out in this study with respect to the total time needed by subjects to complete the task, confirming hypothesis H1 in this regard. The fact that, especially for a higher total number of stories (>6), the 360° and CGI binaural experimental conditions perform better, hence in terms of less task completion time needed, again confirms the positive impact of a more detailed audiovisual representation of the virtual environment on task performance. This is closely related to differences in the auditory and visual representation of the specific 360° and CGI binaural experimental conditions compared to those used in the study by Ahrens et al. (2019). In particular, the lack of lip-sync for the semi-transparent avatar silhouettes in the study by Ahrens et al. (2019) was likely a key factor. In contrast, the 360° binaural experimental condition in this study included lip-sync with corresponding recorded videos for each talker, which likely contributed to the higher task performance observed. For the CGI binaural experimental condition, where lip-sync was missing, the improved task performance compared to the study by Ahrens et al. (2019) can primarily be attributed to changes in the arrangement to a full circle with only one possible distance between the listener and the sources, instead of the semi-circular arrangement with 21 speakers around the listener in 30° steps with three possible distances between the listener and the sources used in Ahrens et al. (2019).

As shown in Figure 4, especially starting from a higher number than seven simultaneously active talkers, participants across all experimental conditions tend to use nearly the entire 120 s time limit. That finding suggests that the time limit may have been too short and perhaps had an influence on the results.

Although the experimental design of the experiments conducted in this study is somewhat different from the experiment by Ahrens et al. (2019), the overall trend in task performance changes, hence in terms of the percentage of correctly assigned stories and total time needed, with an increasing total number of stories presented simultaneously, is still comparable to the study by Ahrens et al. (2019). For a number of 10 simultaneously presented stories, still about 40% of the stories could be assigned correctly, at least for the 360° binaural and CGI binaural experimental conditions. This suggests that it may be possible to extend the paradigm in the future, potentially increasing the total number of simultaneously presented stories as well as the total number of speakers while also increasing the total time available beyond 120 s. The finding that an increase in simultaneous speakers in the scene was accompanied by an increase in total time needed is in line with the results of the study in Slomianka et al. (2024), where the authors stated that an increased scene complexity was accompanied by an extended search period.

### 5.2 Influence of total number of stories and experimental condition on task performance and mental load

With RQ2, cf. Section 1, it was aimed to investigate whether the total number of stories presented simultaneously, the audio condition (binaural vs. diotic), and the visual IVE representation (360° video vs. CGI) impact task performance and mental load. In H2, cf. Section 1, it was hypothesized that an increasing total number of stories presented simultaneously leads to decreasing task performance and increasing mental load, with stronger effects observed in diotic compared to binaural audio conditions and in the CGI representation compared to 360° video. As indicated by the results in the previous section, the total number of simultaneously presented stories appears to have a clear negative impact on task performance, which is also reflected in the increasing mental load of the subjects. This is visible from the percentage of correctly assigned stories, the total time needed, and the results from the NASA RTLX and effort scaling questionnaires related to the mental load of the subjects. This is particularly visible in Figure 5, where stronger effects—and consequently a higher overall mental load across all dimensions of the NASA RTLX and effort scaling question—are observed for the 360° diotic compared to the 360° binaural and CGI binaural experimental conditions, thereby confirming H2. Furthermore, the used experimental condition has, for certain higher numbers of stores, a significant influence on task performance and the mental load. It can also be concluded that across all three experimental conditions, the 360° diotic experimental condition is the one leading to the worst performance and highest load for participants, again confirming hypothesis H2. This is indicated by task performance, which is measured by the percentage of correctly assigned stories and the total time required per total number of simultaneously presented stories. Additionally, it is reflected in terms of mental load, specifically NASA RTLX and listening effort scores. This finding is in line with the results of the study by Rungta et al. (2018), where the authors also found binaural listening to outperform diotic listening. This can be related to the lack of localization cues and reduced aural scene-analysis ability in the diotic audio presentation, which made it hard for participants to distinguish between the individual audio signals of the presented stories.

One key factor that allowed subjects to partially assign the correct stories to the corresponding speakers was audiovisual integration, more specifically lip synchronization, which was intrinsically given for both 360° experimental conditions. This enabled participants to better map the active speakers to the correct stories. Consequently, in terms of task performance, comparing 360° binaural and CGI binaural shows that 360° binaural outperforms CGI binaural as an experimental condition, again confirming hypothesis H2. In the case of the CGI binaural experimental condition, lip synchronization of the avatars was not provided; hence, except for gender, the listener had no visual indicator of who was speaking in the virtual room and had to rely solely on their auditory perception. This apparently led to partially significant differences in terms of task performance, but not in terms of the different dimensions of NASA RTLX and listening effort between these two tests. All in all, it can be stated that in terms of the task performance, that is, total time needed and percentage of correctly assigned stories, and mental load, the CGI binaural experimental condition lies “in-between” the 360° diotic and the 360° binaural experimental conditions.

### 5.3 Influence of experimental condition on user behavior

With RQ3, cf. Section 1, it was aimed to investigate to what extent the user behavior, in terms of head movements (e.g., proportion of time spent watching active speakers, total yaw degrees explored, total number of yaw direction changes), is different across audio conditions (binaural vs. diotic) and visual IVE representations (360° video vs. CGI). In H3, cf. Section 1, it was hypothesized that head movement patterns would differ between binaural and diotic audio conditions, as well as between 360° video and CGI representations. As already shown, there are some influences of the test paradigm on the user behavior, for example, regarding the time participants spent looking at the direction of the active speakers. It turned out that, compared to the other two experimental conditions, in CGI binaural people tend to watch significantly less often the areas of the IVE where active speakers are sitting, partially confirming hypothesis H3. This is assumed to be related to the fact that lip synchronization was missing for the CGI binaural experimental condition, and also no other visual indications were given that a speaker was active, leading to a less “focused” viewing behavior than for the 360° experimental conditions, where lip synchronization and generally visibly active speaking were presented. In turn, for the CGI experimental condition, visual information did not help much, and hence looking at active speakers is reduced, as it is unclear who is an active speaker. For the two 360° experimental conditions, both visual and auditory cues seem to be used, regardless of the acoustic condition. Moreover, the added audio localization in the binaural experimental condition enhances overall task performance and increases the proportion of time spent watching active speakers. Hence, in the case of the CGI binaural experimental condition, subjects needed to rely only on auditory information to map the stories to the respective speakers.

It is interesting to note that between the 360° diotic and binaural experimental conditions, no significant differences could be found, falsifying hypothesis H3, at least when comparing the 360° diotic and binaural experimental conditions directly. Hence, for these two tests, visual feedback in the form of lip movements was the main reason for subjects to look in the direction of the active speakers of the virtual scene. If lip-sync and visual activity are present in the 360° experimental conditions, this will be mainly used, as shown in Figure 6, basically different for CGI. If binaural information can be used, people will do so, as shown in Figure 8, as these help support auditory localization and scene analysis (diotic vs. binaural). In addition, participants tend to watch the parts where active speakers are sitting more frequently, with an increasing total number of stories presented simultaneously. The reasons for this are that with an increasing total number of stories, subjects seem to focus even more on the completion of the task because they spend more time watching the “relevant” parts of the virtual environment, hence the active speakers. The fact that there are significant differences between the CGI binaural and the 360° experimental conditions supports this, as these “relevant” parts were more difficult to identify for the CGI binaural experimental condition. Another reason contributing to this behavior could be that with fewer stories presented simultaneously, the possibility of observing areas where no active speakers are sitting is greater. In contrast, for a higher number of simultaneously presented stories, the possibility of watching areas where active speakers are sitting is also higher.

Another indicator for active exploration of the scene is the total amount of yaw degrees explored. However, except for a few cases of stories presented simultaneously, no significant differences were measured between the different experimental conditions, falsifying hypothesis H3 in this regard. Only a few cases of stories presented simultaneously showed significant differences, primarily between the 360° diotic and the CGI binaural experimental conditions, while exploration was the least in the 360° diotic experimental condition for a larger number of stories presented simultaneously. The assumed reason for this is that for the 360° diotic experimental condition, participants at some point did not really explore the scene anymore but rather focused on understanding different speakers to identify the themes of the stories told, leading to a lower overall yaw exploration of the scene. Another interpretation of that observation may be that for the 360° diotic experimental condition, the task gets so complicated that at some point, no efforts seem to be made to further explore the scene, and the total amount of yaw degrees explored simply ceases to increase. That is also visible from the lower number of yaw direction changes per second for a higher amount of stories presented simultaneously, shown in Figure 9. For the other two experimental conditions, 360° and CGI binaural, head rotations still seem to help to find active speakers, even for a higher number of stories presented simultaneously. For the CGI binaural experimental condition, for a few cases of stories presented simultaneously, participants explored the scene significantly more than in the 360° diotic case. This is likely again related to the fact that in the CGI binaural experimental condition, due to the missing visual information of who is talking in the room, subjects needed to explore the virtual environment more actively to recognize the active speakers. A reason for that could be a higher amount of binaural cueing movements for the CGI binaural test, as described in the study by Kim et al. (2013). This is also shown in Figure 7 as a general trend, at least starting from six stories presented simultaneously. Generally, it can be observed that as the total number of stories increases, the total amount of yaw degrees explored also increases, at least until a specific total number of stories is presented. This is in line with the results of the study in Slomianka et al. (2024), where the authors stated that the increase in scene complexity was accompanied by an increase in head and eye movements. An obvious reason for this is that with a higher total number of stories presented, the task complexity increases; hence, participants spent more time in the specific scene, which leads to more accumulated exploration. Another reason likely is that with a higher number of presented stories, participants tend to more actively “scan” the scene to find the corresponding speaker-story mappings.

As shown in Figure 7, starting from a number of seven stories presented simultaneously, the amount of yaw degrees explored seems to stay at one level or even decrease, as for example for the 360° diotic test condition. One reason for this could be that with a higher number of stories presented, when trying to accurately map the stories to their respective talkers, subjects tend to focus on individual speakers for a longer time rather than switching their attention between multiple speakers. That aligns with the results of the study in Slomianka et al. (2024), where the reduction in average head speed was likely caused by an increase in average fixation duration as the number of speakers grew and reverberation increased, until reaching a specific threshold of seven speakers. In our experiments, this tendency is especially visible for the 360° diotic experimental condition.

The last aspect that is also relevant for analyzing user behavior is the total number of yaw direction changes, which can be seen as an indicator of binaural cueing through active head rotations, as studied in Kim et al. (2013).

From Figure 8, it can be observed that with a higher number of stories presented simultaneously, the total number of yaw direction changes increases. A reason for this could be that participants tend to use a recurring pattern of left-right moving head rotations to better localize which speaker tells which story. This behavior appears to be more distinct for the experimental conditions using binaural audio, even though the differences are only getting significant for a few total numbers of stories presented simultaneously, falsifying hypothesis H3 in this regard. In addition to this, from Figure 9, it can be seen that with a higher number of stories presented simultaneously, the number of yaw direction changes per second decreases. As shown in Table 6, the difference is predominantly significant between the 360° diotic and binaural experimental conditions across nearly all levels of simultaneously presented stories, confirming hypothesis H3 in this regard. Similar to the total number of yaw direction changes, the difference between the 360° diotic and CGI binaural tests is only significant for nine and 10 simultaneously presented stories. The results from Figures 8, 9 may appear contradictory, as the total number of yaw direction changes could be highly correlated with the total time participants needed to complete the task. Additionally, the number of yaw direction changes per second is more strongly correlated with the percentage of correctly assigned stories (*PCC* = 0.43) than the total number of yaw direction changes (*PCC* = −0.18). This suggests that, in relation to task performance, the number of yaw direction changes per second may be a more relevant measure. Similarly to the total amount of yaw degrees explored, it is also visible that after a certain total number of stories presented simultaneously, the total number of yaw direction changes and also the number of yaw direction changes per second stays at one level or even decrease, at least for the 360° diotic experimental condition. This is particularly illustrated in Figure 9 and is likely related to the same reason mentioned earlier: with more active speakers, subjects tend to concentrate on individual speakers for a longer duration rather than shifting their attention between multiple speakers, which may lead to a more detailed exploration of the scene or a partial abandonment of the task.

It is possible that the 120 s task limit influenced subsequent measures of head rotation behavior, such as the proportion of time spent watching active speakers or the total number of yaw direction changes. However, because all participants faced the same time limit, its overall impact on these measures is assumed to be consistent and is therefore considered as minimal. It is likely more related to personality traits, such as noise sensitivity, which may influence head rotation behavior. As indicated in Section 4, noise sensitivity scores are consistent across the three participant groups of the conducted experiments. Therefore, these aspects are not discussed further in this article.

### 5.4 Influence of experimental condition on other QoE-related constructs for IVEs

The last research question, RQ4, cf. Section 1, targeted to investigate the extent to which differences in constructs related to Quality of Experience (QoE) for IVEs, such as simulator sickness and presence, exist across different audio conditions (binaural vs. diotic) and visual IVE representations (360° video vs. CGI). In H4, cf. Section 1, it was hypothesized that simulator sickness levels (before and after the experiment) will not differ between audio conditions (binaural vs. diotic) or visual IVE representations (360° video vs. CGI), while differences are expected in the sense of presence, with higher levels of presence reported for binaural audio conditions and 360° video representations.

From the analysis of the SSQ scores, it can be seen that the time of assessment, that is, whether subjects answer the questionnaire before or after the test, does not have a significant influence on the simulator sickness of the participants. This indicates that the use of IVEs, across all three tested experimental conditions, did not significantly affect simulator sickness in the subjects, thereby confirming hypothesis H4. The three different instances of IVEs can only be used to evaluate audiovisual scene analysis in a virtual classroom scenario if they do not evoke a significant amount of simulator sickness, which was the case for our tests. Compared to previous studies on simulator sickness for 360° videos, such as the one by Singla et al. (2017), where total score values between 30 and 40 are reported, the total scores obtained for the three experimental conditions are rather low. The generally low SSQ values both before and after the test, which is inherently positive, may partly explain why some SSQ dimensions in Figure 10 are rated higher before exposure to the specific experimental condition than after. Since the 95% CIs mostly overlap in these cases, indicating no statistically meaningful difference, and considering the overall low SSQ values suggesting minimal simulator sickness, the observed phenomenon of slightly higher values before the test may reflect measurement variability or participants' overestimation of their symptoms prior to the experiment.

The IPQ was used to assess the impact of the experimental condition on the perceived presence of participants. Except for the statistically significant difference between the 360° binaural experimental condition and the CGI binaural experimental condition for the experienced realism dimension, where 360° binaural was higher, no further statistically significant differences were found; hence, hypothesis H4 is partially falsified, as differences in the sense of presence can only be reported in the experienced realism dimension between the two mentioned experimental conditions. This is probably due to the more natural look of the 360° video scene, leading to a higher realism dimension score than for the CGI binaural experimental condition. However, subjects perceived similar general presence, spatial presence, and involvement scores for the 360° and CGI binaural experimental conditions. From the results, it can also be concluded that the auditory condition did not appear to have a significant impact on the perceived presence of the participant, although the mean scores of the 360° binaural test appear to be a bit higher than those of the 360° diotic experimental condition.

### 5.5 Limitations of this study and future studies

Although scenarios involving a large number of simultaneous speakers within a single scene, such as the paradigm proposed in this study, might represent an exaggerated classroom scenario, it is important to emphasize that ecological validity was not the primary goal of this study. Rather, the aim was to systematically investigate audiovisual interaction effects using a controlled psychometric approach in scenarios with good speech intelligibility, clearly measurable indicators of cognitive performance, and increased visual scene complexity compared to the original paradigm by Ahrens et al. (2019). Consequently, although real-world classroom group work settings typically involve fewer simultaneously active speakers and clearer spatial clustering, the current approach intentionally simplifies spatial clustering by systematically varying the number of simultaneously active speakers as an appropriate independent variable (cf. Ahrens et al., 2019; Ahrens and Lund, 2022). Future work could incorporate more realistic group clustering and additional spatial audio cues, such as noise, to further investigate the influence of these independent variables in more ecologically valid scenarios. However, developing a more realistic and child-appropriate collaborative group work and discussion scenario remains technically challenging, particularly with respect to audiovisual recordings and the overall IVE setup.

Beyond the issues of ecological validity and scene realism discussed above, it is also important to address certain methodological limitations regarding the experimental setup. One limitation of this study is that the maximum total number of simultaneously presented stories is restricted to 10. Therefore, an aspect of future studies could be to increase this number, even though it would only be meaningful for experimental conditions with binaural audio, as for the 360° diotic experimental condition, a total number of 10 stories already leads to a very low mean value of correctly assigned stories (11.2%). An increase in the total number of active speakers could provide more insights into user exploration behavior, such as the total yaw degrees explored and the overall number of yaw directional changes. It would be helpful to understand how these measures, as well as others like the percentage of correctly assigned stories and the time required, would change with a greater number of active speakers. In that case, tests with an increased time window greater than the 120 s used in this study, or even without a time limit, should also be considered. This is especially important given the finding that the task completion time may have been too short.

Another limitation of this study is that, for the CGI experimental condition, any form of visual feedback indicating which speaker is active was omitted, such as lip synchronization, for example. It would be interesting to explore how the presence of visual information might affect the dependent variables discussed in this study. Visual information can be either relatively simple, such as blinking silhouettes around the active speakers, or more complex, involving facial expressions and lip synchronization for the avatars, which would require a more sophisticated technical setup. While the 360° video IVE technically allows only three degrees of freedom (DoF) presentation, an interesting direction for future research is to investigate how a six-DoF CGI IVE influences cognitive performance and exploration behavior, such as simulating a group work activity in a classroom setting. One additional approach for the 360° video IVE could also be to insert still 360° images of the speakers instead of videos to better compare with the results of the CGI binaural experimental condition applied in this article.

Furthermore, it would be interesting to know to what extent the repetition of specific configurations of simultaneously active speakers per participant influences dependent variables, such as user behavior and task performance. Another aspect to investigate is the specific behavioral strategy that participants have employed to complete the task. They likely relied on binaural cueing initially, followed by analyzing the lip-sync of active speakers, ultimately mapping the stories to the respective active speakers. Another aspect to investigate is the extent to which additional factors of realism and complexity in the acoustic representation, such as the integration of classroom acoustics, may influence various dependent variables, like presence, user behavior, or task performance.

## Data Availability

The datasets presented in this study can be found in online repositories. The names of the repository/repositories and accession number(s) can be found in the article/Supplementary material.

## Footnotes

^1^ https://github.com/Telecommunication-Telemedia-Assessment/AVT-ECoClass-VR

^2^ https://git.rwth-aachen.de/ita/VAUnity

^3^ https://github.com/Telecommunication-Telemedia-Assessment/AVT-ECoClass-VR-dataset

## References

[B1] Agtzidis I. Startsev M. Dorr M. (2019). “360-degree video gaze behaviour: a ground-truth data set and a classification algorithm for eye movements,” in Proceedings of the 27th ACM International Conference on Multimedia (New York, NY: ACM), 1007–1015. 10.1145/3343031.3350947

[B2] Ahrens A. Lund K. D. (2022). Auditory spatial analysis in reverberant multi-talker environments with congruent and incongruent audio-visual room information. J. Acoust. Soc. Am. 152, 1586–1594. 10.1121/10.0013991 36182305

[B3] Ahrens A. Lund K. D. Dau T. (2019). “Audio-visual scene analysis in reverberant multi-talker environments,” in 23rd International Congress on Acoustics (Berlin: Deutsche Gesellschaft für Akustik eV), 3890–3896.

[B4] Barutchu A. Spence C. (2021). Top-down task-specific determinants of multisensory motor reaction time enhancements and sensory switch costs. Exp. Brain Res. 239, 1021–1034. 10.1007/s00221-020-06014-3 33515085 PMC7943519

[B5] Bronkhorst A. W. (2000). The cocktail party phenomenon: a review of research on speech intelligibility in multiple-talker conditions. Acta Acust. United Acust. 86, 117–128.

[B6] Bronkhorst A. W. (2015). The cocktail-party problem revisited: early processing and selection of multi-talker speech. Attent. Percept. Psychophys. 77, 1465–1487. 10.3758/s13414-015-0882-9 25828463 PMC4469089

[B7] Chen C. Eickhoff C. (2023). SSE: a metric for evaluating search system explainability. arXiv [Preprint]. arXiv:2306.10175. 10.48550/arXiv.2306.10175

[B8] Cherry E. (1953). Some experiments on the recognition of speech, with one and two ears. J. Acoust. Soc. Am. 25, 975–979. 10.1121/1.1907229

[B9] Doyle A.-B. (1973). Listening to distraction: a developmental study of selective attention. J. Exp. Child Psychol. 15, 100–115. 10.1016/0022-0965(73)90134-3 4706960

[B10] EBU Recommendation (2023). Loudness Normalisation and Permitted Maximum Level of Audio Signals. Geneva: EBU Recommendation.

[B11] Fichna S. Biberger T. Seeber B. U. Ewert S. D. (2021). “Effect of acoustic scene complexity and visual scene representation on auditory perception in virtual audio-visual environments,” in 2021 Immersive and 3D Audio: from Architecture to Automotive (I3DA) (IEEE), 1–9. 10.1109/I3DA48870.2021.9610916

[B12] Flägel K. Galler B. Steinhäuser J. Götz K. (2019). The “national aeronautics and space administration-task load index” (NASA-TLX)-an instrument for measuring consultation workload within general practice: evaluation of psychometric properties. Z. Evid. Fortbild. Qual. Gesundhwes. 147, 90–96. 10.1016/j.zefq.2019.10.003 31759889

[B13] Foreman N. (2010). Virtual reality in psychology. Themes Sci. Technol. Educ. 2, 225–252.

[B14] Fremerey S. Breuer C. Leist L. Klatte M. Fels J. Raake A.. (2024). “AVT-ECOCLASS-VR: an open-source audiovisual 360° video and immersive CGI multi-talker dataset to evaluate cognitive performance,” in 2024 16th International Conference on Quality of Multimedia Experience (QoMEX) (Karlshamn: IEEE), 207–213. 10.1109/QoMEX61742.2024.10598262

[B15] Fremerey S. Singla A. Meseberg K. Raake A. (2018). “Avtrack360: an open dataset and software recording people's head rotations watching 360 videos on an HMD,” in Proceedings of the 9th ACM Multimedia Systems Conference (New York, NY: ACM), 403–408. 10.1145/3204949.3208134

[B16] Gardner W. G. Martin K. D. (1995). Hrtf measurements of a kemar. J. Acoust. Soc. Am. 97, 3907–3908. 10.1121/1.412407

[B17] Georgsson M. (2019). “NASA RTLX as a novel assessment for determining cognitive load and user acceptance of expert and user-based evaluation methods exemplified through a mhealth diabetes self-management application evaluation,” in pHealth 2019 (London: IOS Press), 185–190. 10.3233/978-1-61499-975-1-185 31156113

[B18] Gonzalez-Franco M. Maselli A. Florencio D. Smolyanskiy N. Zhang Z. (2017). Concurrent talking in immersive virtual reality: on the dominance of visual speech cues. Sci. Rep. 7:3817. 10.1038/s41598-017-04201-x 28630450 PMC5476615

[B19] Göring S. Rao R. Fremerey S. Raake A. (2021). “AVrate Voyager: an open source online testing platform,” in 2021 IEEE 23st International Workshop on Multimedia Signal Processing (MMSP) (Tampere: IEEE), 1–6. 10.1109/MMSP53017.2021.9733561

[B20] Helton W. S. Jackson K. M. Näswall, K. Humphrey B. (2022). “The national aviation and space agency task load index (NASA-TLX): does it need updating?” in Proceedings of the Human Factors and Ergonomics Society Annual Meeting, Volume 66 (Los Angeles, CA: SAGE Publications), 1245–1249. 10.1177/1071181322661370

[B21] Holmes E. Kitterick P. T. Summerfield A. Q. (2016). Eeg activity evoked in preparation for multi-talker listening by adults and children. Hear. Res. 336, 83–100. 10.1016/j.heares.2016.04.007 27178442

[B22] HTC Corporation (2021). Important Safety Information – *Read Before Use*. Available online at: https://dl.vive.com/safety-guide/vive-pro/pro2-hmd/91H03228-02M_RevB.pdf (accessed Novmber 24, 2023).

[B23] IHTA RWTH Aachen University. (2024). Virtual Acoustics-A Real-Time Auralization Framework for Scientific Research. Available online at: http://www.virtualacoustics.org (accessed August 27, 2024).

[B24] Ishihara S. (2009). The Series of Plates Designed as a Test for Color Deficiency. Tokyo: Kanehara Trading Inc.

[B25] Josupeit A. Hohmann V. (2017). Modeling speech localization, talker identification, and word recognition in a multi-talker setting. J. Acoust. Soc. Am. 142, 35–54. 10.1121/1.4990375 28764452

[B26] Kay M. Elkin L. Higgins J. Wobbrock J. (2021). Artool: Aligned Rank Transform for Nonparametric Factorial ANOVAs, r package version 0.11.0. 10.5281/zenodo.594511

[B27] Kennedy R. S. Lane N. E. Berbaum K. S. Lilienthal M. G. (1993). Simulator sickness questionnaire: an enhanced method for quantifying simulator sickness. Int. J. Aviat. Psychol. 3, 203–220. 10.1207/s15327108ijap0303_327885969

[B28] Kim C. Mason R. Brookes T. (2013). Head movements made by listeners in experimental and real-life listening activities. J. Audio Eng. Soc. 61, 425–438.

[B29] Kishline L. R. Colburn S. W. Robinson P. W. (2020). A multimedia speech corpus for audio visual research in virtual reality (l). J. Acoust. Soc. Am. 148, 492–495. 10.1121/10.0001670 32873016

[B30] Koch I. Lawo V. Fels J. Vorländer M. (2011). Switching in the cocktail party: exploring intentional control of auditory selective attention. J. Exp. Psychol.: Hum. Percept. Perform. 37:1140. 10.1037/a0022189 21553997

[B31] Lawo V. Fels J. Oberem J. Koch I. (2014). Intentional attention switching in dichotic listening: exploring the efficiency of nonspatial and spatial selection. Q. J. Expe. Psychol. 67, 2010–2024. 10.1080/17470218.2014.898079 25248101

[B32] Lin J.-W. Duh H. B.-L. Parker D. E. Abi-Rached H. Furness T. A. (2002). “Effects of field of view on presence, enjoyment, memory, and simulator sickness in a virtual environment,” in Proceedings IEEE Virtual Reality 2002 (Orlando, FL: IEEE), 164–171. 10.1109/VR.2002.996519

[B33] Oberem J. Koch I. Fels J. (2017). Intentional switching in auditory selective attention: exploring age-related effects in a spatial setup requiring speech perception. Acta Psychol. 177, 36–43. 10.1016/j.actpsy.2017.04.008 28456098

[B34] Oberem J. Lawo V. Koch I. Fels J. (2014). Intentional switching in auditory selective attention: exploring different binaural reproduction methods in an anechoic chamber. Acta Acust. United Acust. 100, 1139–1148. 10.3813/AAA.918793

[B35] Oberem J. Seibold J. Koch I. Fels J. (2018). Intentional switching in auditory selective attention: exploring attention shifts with different reverberation times. Hear. Res. 359, 32–39. 10.1016/j.heares.2017.12.013 29305038

[B36] Owens A. Efros A. A. (2018). “Audio-visual scene analysis with self-supervised multisensory features,” in Proceedings of the European conference on computer vision (ECCV) (Cham: Springer), 631–648. 10.1007/978-3-030-01231-1_39

[B37] Parkin S. Driss S. Krol K. Sasse M. A. (2016). “Assessing the user experience of password reset policies in a university,” in Technology and Practice of Passwords: 9th International Conference, PASSWORDS 2015, Cambridge, UK, December 7-9, 2015, Proceedings 9 (Cham: Springer), 21–38. 10.1007/978-3-319-29938-9_2

[B38] Pro Visu Foundation (2023). Tests - *ProVisu*. Available online at: https://www.provisu.ch/en/screening/tests (accessed November 24, 2023).

[B39] Röer J. P. Bell R. Körner U. Buchner A. (2018). Equivalent auditory distraction in children and adults. J. Exp. Child Psychol. 172, 41–58. 10.1016/j.jecp.2018.02.005 29574236

[B40] Rossi D. Aricò P. Di Flumeri G. Ronca V. Giorgi A. Vozzi A.. (2024). Analysis of head micromovements and body posture for vigilance decrement assessment. Appl. Sci. 14:1810. 10.3390/app14051810

[B41] Rueda M. R. Fan J. McCandliss B. D. Halparin J. D. Gruber D. B. Lercari L. P.. (2004). Development of attentional networks in childhood. Neuropsychologia 42, 1029–1040. 10.1016/j.neuropsychologia.2003.12.012 15093142

[B42] Rungta A. Rewkowski N. Schissler C. Robinson P. Mehra R. Manocha D.. (2018). “Effects of virtual acoustics on target-word identification performance in multi-talker environments,” in Proceedings of the 15th ACM Symposium on Applied Perception (New York, NY: ACM), 1–8. 10.1145/3225153.3225166

[B43] Said S. Gozdzik M. Roche T. R. Braun J. Rössler J. Kaserer A.. (2020). Validation of the raw national aeronautics and space administration task load index (NASA-TLX) questionnaire to assess perceived workload in patient monitoring tasks: pooled analysis study using mixed models. J. Med. Internet Res. 22:e19472. 10.2196/19472 32780712 PMC7506540

[B44] Schmutz P. Heinz S. Métrailler Y. Opwis K. (2009). Cognitive load in ecommerce applications-measurement and effects on user satisfaction. Adv. Hum. Comput. Interact. 2009, 121494-1. 10.1155/2009/121494

[B45] Schnall S. Hedge C. Weaver R. (2012). The immersive virtual environment of the digital fulldome: considerations of relevant psychological processes. Int. J. Hum. Comput. Stud. 70, 561–575. 10.1016/j.ijhcs.2012.04.001

[B46] Schubert T. Friedmann F. Regenbrecht H. (2001). The experience of presence: factor analytic insights. Presence 10, 266–281. 10.1162/105474601300343603

[B47] Sevinc V. Berkman M. I. (2020). Psychometric evaluation of simulator sickness questionnaire and its variants as a measure of cybersickness in consumer virtual environments. Appl. Ergon. 82:102958. 10.1016/j.apergo.2019.102958 31563798

[B48] Shinn-Cunningham B. G. (2008). Object-based auditory and visual attention. Trends Cogn. Sci. 12, 182–186. 10.1016/j.tics.2008.02.003 18396091 PMC2699558

[B49] Singla A. Fremerey S. Robitza W. Raake A. (2017). “Measuring and comparing qoe and simulator sickness of omnidirectional videos in different head mounted displays,” in 2017 Ninth international conference on quality of multimedia experience (QoMEX) (Erfurt: IEEE), 1–6. 10.1109/QoMEX.2017.7965658

[B50] Slomianka V. Dau T. Ahrens A. (2024). Acoustic scene complexity affects motion behavior during speech perception in audio-visual multi-talker virtual environments. Sci. Rep. 14:19028. 10.1038/s41598-024-70026-0 39152193 PMC11329770

[B51] Spence C. Driver J. (1997). On measuring selective attention to an expected sensory modality. Percept. Psychophys. 59, 389–403. 10.3758/BF03211906 9136269

[B52] Stecker G. C. (2019). Using virtual reality to assess auditory performance. Hear. J. 72, 20–22. 10.1097/01.HJ.0000558464.75151.52 34113058 PMC8188812

[B53] Stecker G. C. Moore T. M. Folkerts M. Zotkin D. Duraiswami R. (2018). “Toward objective measures of auditory co-immersion in virtual and augmented reality,” in Audio Engineering Society Conference: 2018 AES International Conference on Audio for Virtual and Augmented Reality (Redmond, WA: Audio Engineering Society).

[B54] Van Esch T. E. Kollmeier B. Vormann M. Lyzenga J. Houtgast T. Hällgren M.. (2013). Evaluation of the preliminary auditory profile test battery in an international multi-centre study. Int. J. Audiol. 52, 305–321. 10.3109/14992027.2012.759665 23570289

[B55] Vollmer L. J. Ermert C. A. Fels J. (2023). “Temporal mismatch effects in short-term memory of audio-visually presented spoken digits,” in Proceedings *of the 10th Convention of the European Acoustics Association, Forum Acusticum 2023* (Turin: European Acoustics Association), 5017–5020. 10.61782/fa.2023.1050

[B56] Wobbrock J. O. Findlater L. Gergle D. Higgins J. J. (2011). “The aligned rank transform for nonparametric factorial analyses using only ANOVA procedures,” in Proceedings of the SIGCHI conference on human factors in computing systems (New York, NY: ACM), 143–146. 10.1145/1978942.1978963

[B57] Zimmer K. Ellermeier W. (1997). A German version of weinstein's noise sensitivity scale. Z Lärmbekämpfung 44, 107–110.

