# Supplementary material for: Speaker-story mapping as a method to evaluate audiovisual scene analysis in a virtual classroom scenario
Source: Front Psychol. 2025 Jun 10;16:1520630. doi: 10.3389/fpsyg.2025.1520630 (PMC12185469; doi:10.3389/fpsyg.2025.1520630)
Supplement: Supplementary file 1 [file Data_Sheet_1.pdf]

Supplementary Material

1 SUPPLEMENTARY DATA

Supplementary Material should be uploaded separately on submission. Please include any supplementary data, figures and/or tables. All supplementary files are deposited to FigShare for permanent storage and receive a DOI.

Supplementary material is not typeset so please ensure that all information is clearly presented, the appropriate caption is included in the file and not in the manuscript, and that the style conforms to the rest of the article. To avoid discrepancies between the published article and the supplementary material, please do not add the title, author list, affiliations or correspondence in the supplementary files.

1.1 Distribution of Noise Sensitivity

Based on Zimmer and Ellermeier (1997), Weinstein noise sensitivity item mean values were calculated per test as means of the 21 questionnaire items asked from each subject. In the study from Zimmer and Ellermeier (1997), 275 subjects needed to fill in the questionnaire, whether the mean of the Weinstein noise sensitivity item mean values resulted in 3.04. The results reveal that in all three subjective tests, the means of the Weinstein noise sensitivity item mean values are very similar, namely 2.12 for the 360° diotic test, 2.17 for the 360° binaural test, and 2.29 for the CGI binaural test. That indicates that the noise sensitivity is consistent across the different subject groups in the various experiments conducted. A boxplot of the Weinstein noise sensitivity item mean values can be found in Supplementary Figure S4.

1.2 Influence of Total Number of Stories and Experimental Condition on Percentage of Correctly Assigned Stories

A nonparametric mixed ANOVA has been applied to the transformed data. The results revealed that the interaction effect between the experimental condition and the total number of stories presented simultaneously is statistically significant $(F(16, 736) = 23.78, p = 2.74 * 10^{-56}$. That means that the influence of the total number of stories presented on the percentage of correctly assigned stories is not the same across the three different auditory and visual experimental conditions. There was a significant effect of the total number of stories presented simultaneously on the percentage of correctly assigned stories $(F(8, 736) = 245.07, p = 9.67 * 10^{-202})$. Furthermore, there was a significant effect of the experimental condition used on the percentage of correctly assigned stories $(F(2, 92) = 38.2, p = 8.37 * 10^{-13})$.

1.3 Influence of Total Number of Stories and Experimental Condition on Task Completion Time

A nonparametric mixed ANOVA has been applied to the transformed data. The results revealed that the interaction effect between the experimental condition and the total time needed is statistically significant $(F(16, 736) = 16.82, p = 3.26 * 10^{-40})$. That means that the influence of the total number of stories presented on the total time needed varies between the three different auditory and visual experimental conditions. There was a significant effect of the total number of stories presented simultaneously on the total time needed $(F(8, 736) = 337.8, p = 1.75 * 10^{-240})$. Furthermore, there was a significant effect of the experimental condition used on the total time needed $(F(2, 92) = 36.25, p = 2.46 * 10^{-12})$.

1.4 Influence of Total Number of Stories and Experimental Condition on Proportion of Time Spent Watching Active Speakers

A nonparametric mixed ANOVA has been applied to the transformed data. The results show that the interaction effect between the experimental condition and the proportion of time spent watching active speakers is not statistically significant $(F(16, 736) = 1.52, p = 0.088)$. That means that the impact of the total number of stories presented on the proportion of time spent watching active speakers does not differ significantly between different auditory and visual experimental conditions. There was a significant effect of the total number of stories presented simultaneously on the proportion of time spent watching active speakers $(F(8, 736) = 56.48, p = 1.49 * 10^{-71})$. Furthermore, there was a significant effect of the experimental condition used on the proportion of time spent watching active speakers $(F(2, 92) = 62.71, p = 6.58 * 10^{-18})$.

1.5 Influence of Total Number of Stories and Experimental Condition on Total Yaw Degrees Explored

A nonparametric mixed ANOVA has been applied to the transformed data. The results revealed an interaction effect between the conducted test and the total yaw degrees explored that is statistically significant $(F(16, 736) = 6.61, p = 3.98 * 10^{-14})$. That means that the influence of the total number of stories presented on the total yaw degrees explored differs significantly between different auditory and visual experimental conditions. There was a significant effect of the total number of stories presented simultaneously on the total yaw degrees explored $(F(8, 736) = 38.32, p = 4.92 * 10^{-51})$. Furthermore, there was a significant effect of the experimental condition used on the total yaw degrees explored $(F(2, 92) = 5.33, p = 0.006)$.

1.6 Influence of Total Number of Stories and Experimental Condition on Yaw Direction Changes

A nonparametric mixed ANOVA has been applied to the transformed data with respect to the total number of yaw direction changes. The results revealed that the interaction effect between the conducted test and the total number of yaw direction changes is statistically significant $(F(16, 736) = 6.54, p = 5.93 * 10^{-14})$. That means that the influence of the total number of stories presented on the total number of yaw direction changes differs significantly between different auditory and visual experimental conditions. There was a significant effect of the total number of stories presented simultaneously on the total number of yaw direction changes $(F(8, 736) = 77.1, p = 4.23 * 10^{-92})$. Furthermore, there was no significant effect of the experimental condition used on the total number of yaw direction changes $(F(2, 92) = 2.78, p = 0.067)$.

A nonparametric mixed ANOVA has also been applied to the transformed data with respect to the number of yaw direction changes per second. The results revealed that the interaction effect between the conducted test and the number of yaw direction changes per second is statistically significant $(F(16, 736) = 2.76, p = 0.0002)$. That means that the influence of the total number of stories presented on the number of yaw direction changes per second differs significantly between different auditory and visual experimental conditions. There was a significant effect of the total number of stories presented simultaneously on the number of yaw direction changes per second $(F(8, 736) = 50.41, p = 5.7 * 10^{-65})$. Furthermore, there was a significant effect of the experimental condition used on the number of yaw direction changes per second $(F(2, 92) = 11.97, p = 2.4 * 10^{-5})$.

2 SUPPLEMENTARY TABLES AND FIGURES

For more information on Supplementary Material and for details on the different file types accepted, please see the Supplementary Material section of the Author Guidelines.

Figures, tables, and images will be published under a Creative Commons CC-BY licence and permission must be obtained for use of copyrighted material from other sources (including re-published/adapted/modified/partial figures and images from the internet). It is the responsibility of the authors to acquire the licenses, to follow any citation instructions requested by third-party rights holders, and cover any supplementary charges.

2.1 Figures

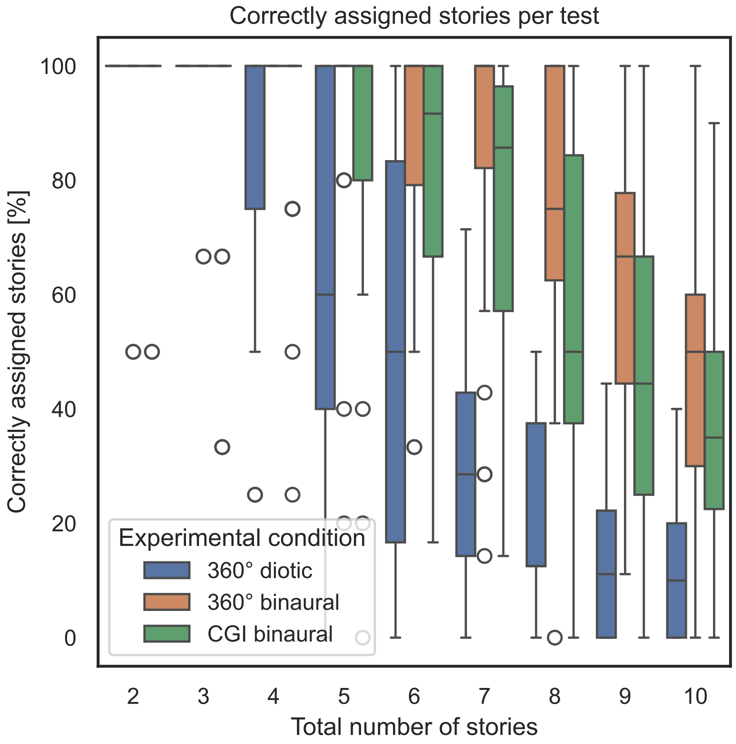

Figure S1. Outlier detection: Total number of stories vs. Percentage of correctly assigned stories. Outliers are represented by the circles.

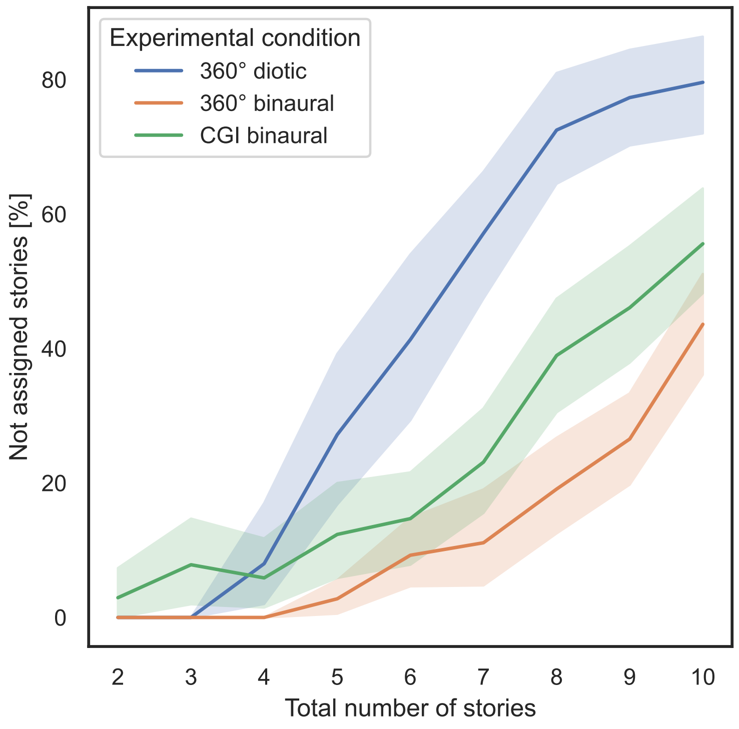

Figure S2. Total number of stories vs. Not assigned stories per test.

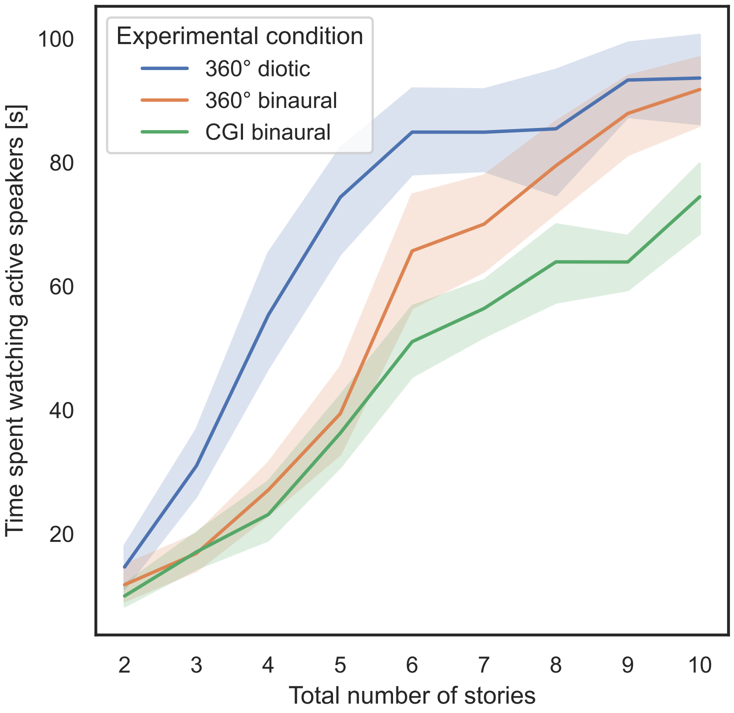

Figure S3. Total number of stories vs. Time spent watching active speakers.

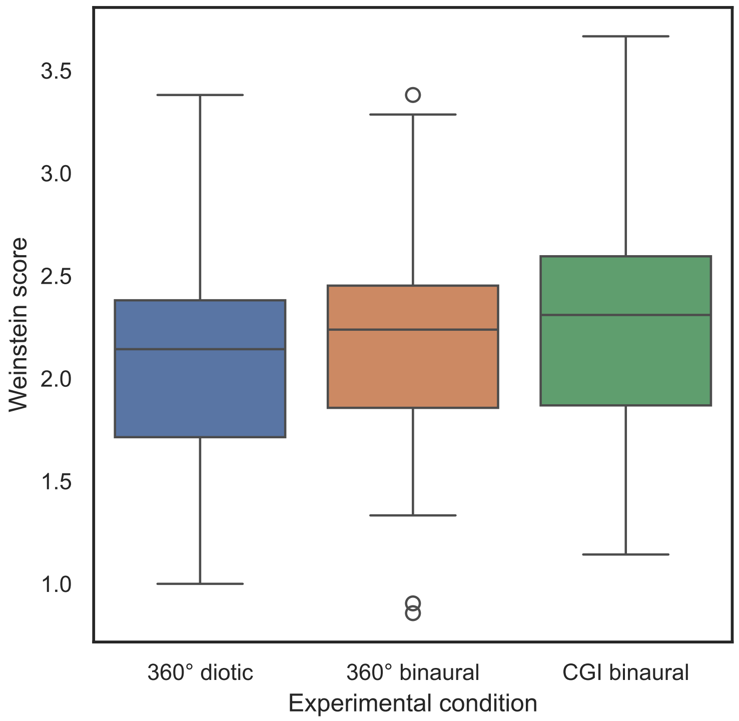

Figure S4. Weinstein questionnaire scores per test.

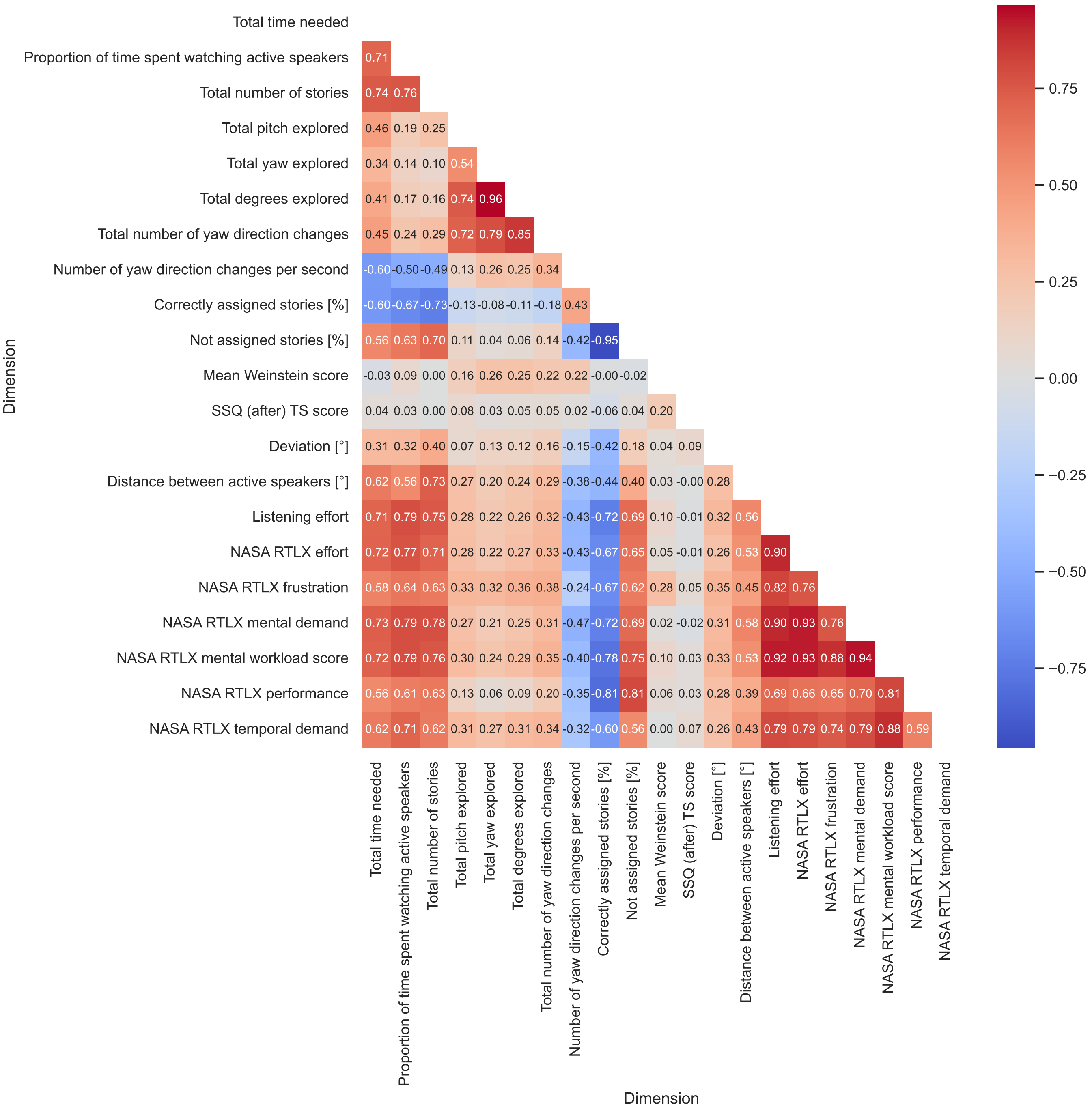

Figure S5. Correlation matrix (all DVs and IVs included), with Pearson's correlation coefficients per pair of variables. From the recorded NASA RTLX values, one unweighted score, called the total NASA RTLX mental workload score, was calculated by averaging the raw data from the five factors.

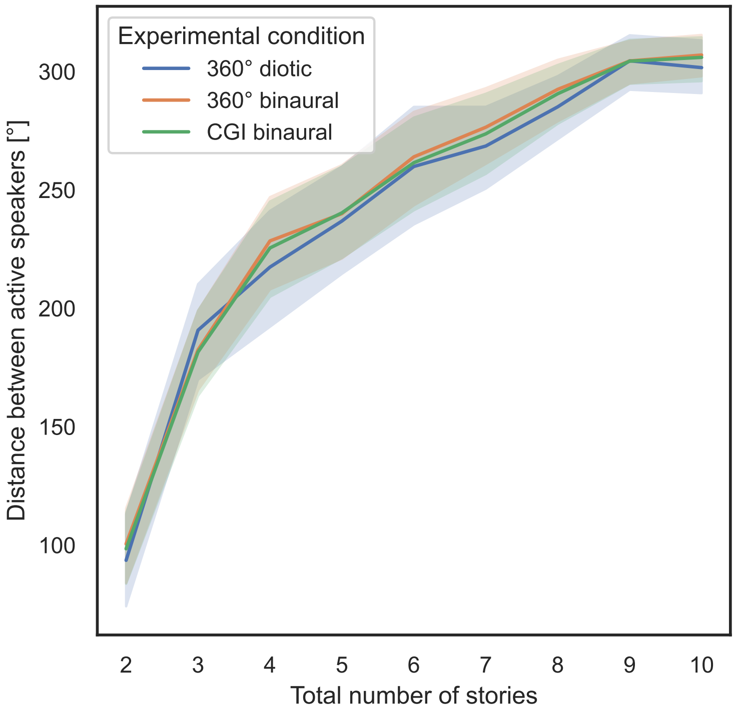

Figure S6. Total number of stories (IV) vs. Distance between active speakers (IV).

2.2 Tables

Contrast	F	p-corr
NRTLX effort * Test	7.77	0.0008
NRTLX effort * Total number of Stories	219.97	$2.05 * 10^{-189}$
NRTLX effort * Interaction	5.04	$5.2 * 10^{-10}$
NRTLX frustration * Test	5.19	0.007
NRTLX frustration * Total number of Stories	146.26	$1.63 * 10^{-146}$
NRTLX frustration * Interaction	3.06	$5.12 * 10^{-5}$
NRTLX mental demand * Test	9.96	0.0001
NRTLX mental demand * Total number of Stories	235.13	$5.69 * 10^{-197}$
NRTLX mental demand * Interaction	4.19	$8.28 * 10^{-8}$
NRTLX performance * Test	12.13	$2.11 * 10^{-5}$
NRTLX performance * Total number of Stories	54.22	$3.88 * 10^{-69}$
NRTLX performance * Interaction	4.68	$4.42 * 10^{-9}$
NRTLX temporal demand * Test	5.99	0.004
NRTLX temporal demand * Total number of Stories	129.4	$7.49 * 10^{-135}$
NRTLX temporal demand * Interaction	3.82	$6.81 * 10^{-7}$
Listening effort * Test	10.95	$5.4 * 10^{-5}$
Listening effort * Total number of Stories	281.28	$5.28 * 10^{-218}$
Listening effort * Interaction	5.45	$4.38 * 10^{-11}$

Table S1. Results from nonparametric mixed ANOVAs applied to computed ART model across all NASA RTLX dimensions, and listening effort. Only significant effects reported.

Contrast	#S	A	B	p-corr
NRTLX effort * Test	-	360° diotic	360° binaural	0.0002
NRTLX effort * Test	-	360° diotic	CGI binaural	0.044
NRTLX effort * Test	-	360° diotic	CGI binaural	0.045
NRTLX effort * #S	4	360° diotic	360° binaural	$9.98 * 10{-}5$
NRTLX effort * #S	5	360° diotic	360° binaural	$4.34 * 10{-}6$
NRTLX effort * #S	5	360° diotic	CGI binaural	0.041
NRTLX effort * #S	6	360° diotic	360° binaural	0.003
NRTLX effort * #S	7	360° diotic	360° binaural	0.021
NRTLX frustration * Test	-	360° diotic	360° binaural	0.002
NRTLX frustration * Test	-	360° diotic	CGI binaural	0.024
NRTLX frustration * #S	4	360° diotic	360° binaural	0.044
NRTLX frustration * #S	5	360° diotic	360° binaural	0.003
NRTLX mental demand * Test	-	360° diotic	360° binaural	0.00002
NRTLX mental demand * Test	-	360° diotic	CGI binaural	0.011
NRTLX mental demand * #S	4	360° diotic	360° binaural	0.003
NRTLX mental demand * #S	5	360° diotic	360° binaural	$1.44 * 10{-}5$
NRTLX mental demand * #S	6	360° diotic	360° binaural	0.002
NRTLX mental demand * #S	7	360° diotic	360° binaural	$8.82 * 10{-}5$
NRTLX mental demand * #S	8	360° diotic	360° binaural	0.0004
NRTLX performance * Test	-	360° diotic	360° binaural	0.00001
NRTLX performance * Test	-	360° diotic	CGI binaural	0.0001
NRTLX performance * #S	5	360° diotic	360° binaural	0.003
NRTLX performance * #S	6	360° diotic	360° binaural	0.013
NRTLX performance * #S	6	360° diotic	CGI binaural	0.021
NRTLX performance * #S	7	360° diotic	360° binaural	$1.58 * 10{-}7$
NRTLX performance * #S	7	360° diotic	CGI binaural	0.0003
NRTLX performance * #S	8	360° diotic	360° binaural	0.0002
NRTLX performance * #S	8	360° diotic	CGI binaural	0.004
NRTLX performance * #S	9	360° diotic	360° binaural	0.003
NRTLX performance * #S	9	360° diotic	CGI binaural	0.005
NRTLX temporal demand * Test	-	360° diotic	360° binaural	0.0008
NRTLX temporal demand * Test	-	360° diotic	CGI binaural	0.044
NRTLX temporal demand * #S	4	360° diotic	360° binaural	0.001
NRTLX temporal demand * #S	5	360° diotic	360° binaural	0.0003
NRTLX temporal demand * #S	6	360° diotic	360° binaural	0.014
Listening effort * Test	-	360° diotic	360° binaural	0.00001
Listening effort * Test	-	360° diotic	CGI binaural	0.003
Listening effort * #S	4	360° diotic	360° binaural	0.005
Listening effort * #S	5	360° diotic	360° binaural	$7.34 * 10{-}8$
Listening effort * #S	5	360° diotic	CGI binaural	0.001
Listening effort * #S	6	360° diotic	360° binaural	$2.29 * 10{-}5$
Listening effort * #S	6	360° diotic	CGI binaural	0.017
Listening effort * #S	7	360° diotic	360° binaural	$6.95 * 10{-}5$
Listening effort * #S	8	360° diotic	360° binaural	0.0007
Listening effort * #S	8	360° diotic	CGI binaural	0.037

Table S2. Results from Bonferroni-corrected contrast tests across all experimental conditions, NASA RTLX dimensions, and listening effort. Only significant effects reported.
#S refers to the total number of stories presented simultaneously, while the experimental condition is specified in “A” and “B”.
